# Breaking down population density into different components to better understand its spatial variation

**DOI:** 10.1186/s12862-021-01809-6

**Published:** 2021-05-11

**Authors:** Mickaël Jacquier, Jean-Michel Vandel, François Léger, Jeanne Duhayer, Sylvia Pardonnet, Ludovic Say, Sébastien Devillard, Sandrine Ruette

**Affiliations:** 1 CNRS, UMR5558 LBBE, Univ Lyon, Université Claude Bernard Lyon 1, 69100 Villeurbanne, France; 2 Office Français de La Biodiversité, Unité-PAD, Montfort, 01330 Birieux, France; 3 Office Français de La Biodiversité, Unité-PAD, 67150 Gerstheim, France

**Keywords:** Population density, Group size, Large-scale, *Meles meles*, *Mustelidae*, Distance sampling, Molecular ecology, Camera trap

## Abstract

**Background:**

Population size and densities are key parameters in both fundamental and applied ecology, as they affect population resilience to density-dependent processes, habitat changes and stochastic events. Efficient management measures or species conservation programs thus require accurate estimates of local population densities across time and space, especially for continuously distributed species. For social species living in groups, population density depends on different components, namely the number of groups and the group size, for which relative variations in space may originate from different environmental factors. Whether resulting spatial variations in density are mostly triggered by one component or the other remains poorly known. Here, we aimed at determining the magnitude of the spatial variation in population densities of a social, group-living species, i.e. the European badger *Meles meles*, in 13 different sites of around 50 km^2^ across France, to decipher whether sett density, group size or proportion of occupied sett variation is the main factor explaining density variation. Besides the intrinsic factors of density variation, we also assessed whether habitat characteristics such as habitat fragmentation, urbanisation, and resource availability, drove both the spatial variation of density components and local population densities.

**Results:**

We proposed a new standardised approach combining use of multiple methods, namely distance sampling for estimating the density of occupied sett clusters, i.e. group density, and camera and hair trapping for genetic identification to determine the mean social group size. The density of adult badgers was on average 3.8 per km^2^ (range 1.7–7.9 per km^2^) and was positively correlated with the density of sett clusters. The density of adult badgers per site was less related to the social group size or to the proportion of occupied sett clusters. Landscape fragmentation also explained the spatial variation of adult badger density, with highly fragmented landscapes supporting lower adult densities. Density components were linked differently to environmental variables.

**Conclusions:**

These results underline the need to break down population density estimates into several components in group-living species to better understand the pattern of temporal and spatial variation in population density, as different components may vary due to different ecological factors.

**Supplementary Information:**

The online version contains supplementary material available at 10.1186/s12862-021-01809-6.

## Background

Estimating population size and density is at the core of both fundamental ecology (e.g. population dynamics over space and time) and applied ecology (e.g. wildlife management). Indeed, density-dependent effects and responses to resource variation, gradual habitat changes or stochastic environments jointly affect population size and density, and ultimately population resilience [1]. Reliable estimates of population size and density in space and/or time are often necessary either to preserve (endangered species such as tigers [2]), control (pests or vectors of disease, for example badgers across Europe [3] and several bat species [4]) or understand the evolutionary ecology of wild populations. Moreover, for species distributed over large ranges, obtaining accurate estimates of global and local abundance in multiple populations and understanding the pattern of their spatial variation are essential to implement appropriate management and conservation measures. For example, spatial variation in population abundance has been linked to the satisfaction of species-specific ecological requirements at local sites [5, 6] or to the location of the site relative to the centre of the species distribution area [5, 7]. However, deriving accurate estimates of population size and density can be difficult. Detecting individuals may be difficult in itself (for cryptic or nocturnal species [8]), and the species may be continuously present over such a large range that its population density greatly vary in space depending on the local environment [9].

When species have a wide spatial distribution, local conditions likely affect local population densities. To understand and predict spatial variation in population density at the large scale, it is crucial to look for ecological factors (e.g. habitat quality) influencing sub-populations at the local scale. Availability of food resources and habitat fragmentation strongly affect local settlement/refuge [10, 11]. The social structure of group-living species may also be complex, influencing both the number of social groups and group size. Local environmental conditions may differently affect group sizes and number of territories, i.e. the number of social groups [12], so that the resulting population density variation may be more or less triggered by variation in both density components. Therefore, to better understand population density variation of widespread social species, a possible approach consist of: (i) estimating group sizes and number of groups at multiple sites, (ii) investigating which component influences population density the most, and (iii) identifying which environmental factors affect the observed densities and their components.

Several methods are commonly used to estimate population density in wildlife ecology, depending on the scale of interest (live trapping combined with capture–mark–recapture analyses, direct observations of individuals through distance sampling methodology, indirect non-invasive data collection methods like detecting signs of presence, genetic tools, and camera traps). Nevertheless, each of these methods presents some limitations and, to optimally choose among them, it is crucial to take into account the ecology of the focus species (e.g. social system, activity patterns, foraging behaviour, etc. [8]). This is particularly important in social species where densities depend not only on territory sizes and the number of groups in a given area, but also on group size [13]. However, even if the mere presence of a group of individuals can be easily detected through direct observations, estimating its size in a particular area can be tedious, as individuals must be visually differentiated, which is not always possible based on phenotype [14]. In that respect, the development of genetic tools (such as microsatellite markers) allows us to circumvent this difficulty and confidently identify individuals based on their genotype. Combining direct methods to localise areas of interest and estimate their density (e.g. territory, sett, or any clue indicating the presence of a group), and indirect methods to differentiate individuals, and therefore count individuals of a given group, offers a promising approach for estimating population density in social species [15].

One example of a social and widely distributed mammal is the European badger *Meles meles*, which occupies a broad range of environments across Europe [16]. Badgers live in territorial and mixed-sex social groups, inhabiting one or several setts in their territories [17]. Using different methodological approaches (see [18] for a synthesis across Europe), various authors have shown that badgers accommodate to contrasting environmental conditions, and that their densities, group size, proportion of breeding setts, and group territory size, vary both among and within regions as a consequence of variation in factors such as the availability of preferred habitat [19]. Nevertheless, to our knowledge, no study has broken down population density into its different components, namely sett density, group size, and percentage of occupied setts, based on a comprehensive set of local populations using up-to-date methodological approaches. Such a standardised approach might be easily exported to other social and group-living species.

In the present study, we aimed at determining badger densities in 13 different areas of metropolitan France, using a robust combination of methodological approaches. In moderate to high density populations, as the territory of a social group can encompass several setts [20–22], we grouped nearby setts together and considered clusters of setts instead of setts. We first estimated the density of sett clusters of each study site using distance sampling [23, 24] on walked transects, and then estimated the proportion of occupied sett clusters (an approximation of the number of social groups in a given area). Second, we determined mean social group size using two non-invasive methods, i.e. camera trapping and hair trapping for genetic identification. These methods have been previously used to estimate mean social group size in badger populations (see for camera trapping [25, 26] and for hair trapping [27, 28]). Finally, we estimated the density of badger as: D = A × B × C/Area, with (A) the number of sett clusters, (B) the proportion of occupied sett clusters, (C) the number of animals per occupied sett cluster, and Area the size of the study site.

While badgers are present throughout metropolitan France in varying abundances across regions [29], we expected contrasting local densities as a result of significant variations in forest cover and human pressure, leading to quantitative and qualitative habitat variation between study sites. We investigated whether spatial variation in population density was mainly due to variation in social group size or in sett cluster density. Besides these intrinsic factors of variation, we also investigated whether habitat characteristics, i.e. the magnitude of forested habitat fragmentation and resource availability, are correlated to the local population density, as shown at the regional scale throughout France [30].

## Results

In each of the 13 study sites, 76.3 to 93.4 km were walked. Over the 13 study sites, realized transect length ranged from 0.4 to 3.2 km (mean = 1.7 km ± 0.4 SD), which was quite similar to planned transects (difference being attributed to walking difficulties on the field mostly due to vegetation cover). A total of 533 setts were detected (mean = 41.0 ± 19.2 SD), among which 231 setts were surveyed to estimate mean badger group sizes. These 533 setts were grouped into 273 sett clusters using the between-setts distance of 500 m (Table 1). Of the 273 sett clusters found, 96 were composed of unoccupied setts (35.2%), 57 included only secondary setts with at least one occupied (i.e. secondary sett clusters, SSC; 20.9%), 82 included at least one occupied main sett without reproduction (i.e. main sett clusters, MSC; 30%) and finally 38 included at least one occupied main sett with reproduction (i.e. main sett clusters with reproduction, MSCR; 13.9%).

**Table 1:** Number of setts detected in the walked transects survey and number used to determine social group size in the 13 study sites (from A to M) in France

Study site	Density of sett clusters estimation					Group size estimation		
	Number of setts detected	Number of sett clusters formed	*p*_SSC_	*p*_MSC_	*p*_MSCR_	Number of setts surveyed	Number of secondary sett clusters with results	Number of main sett clusters with results
A	78	24	0.13	0.13	0.13	19	2	11
B	40	23	0.13	0.26	0.22	27	1	15
C	35	21	0.10	0.14	0.33	20	2	15
D	66	29	0.52	0.17	0.14	20	5	9
E	35	18	0.06	0.39	0.17	16	0	12
F	36	22	0.32	0.23	0.05	17	3	5
G	38	18	0.22	0.17	0.17	9	2	6
H	46	26	0.38	0.27	0.08	16	2	9
I	29	15	0.07	0.47	0.27	20	0	14
J	21	16	0.06	0.25	0.06	16	1	13
K	68	34	0.21	0.41	0.15	20	1	12
L	11	11	0.09	0.82	0.00	14	0	11
M	30	16	0.13	0.56	0.00	17	1	9
Total/mean	533	273	0.18	0.33	0.13	231	20	141

*p*_SSC_, *p*_MSC_, and *p*_MSCR_ correspond respectively to the proportion of occupied (i) secondary sett clusters (SSC), (ii) main sett clusters without reproduction (MSC), and (iii) main sett clusters with reproduction (MSCR) per site

### Sett cluster density

We first modelled a detection function from the histogram of the observed perpendicular distance data (Additional file 1). Among the various detection functions tested, the hazard rate (HR) function had the lowest AIC (Table 2) and fitted well the data (χ^2^ = 1.58, df = 4, p-value = 0.81). The top-ranked model used a post-stratification using the habitat type (i.e. forested or hedgerows sites) of the whole study site SIT (18.6 drop in AIC when compared to pooled data model). Hence, we observed a significantly higher detectability and effective strip width in forested sites than in hedgerow sites (19.7 ± 4.2 m with standard deviation and 10.7 ± 1.2 m, respectively). All other variables tested i.e. habitat type along the transect (HAB), the type of sett cluster (TER), and multiple covariates distance sampling did not improve the model (Table 2).

**Table 2:** Parameters estimates of the seven top-ranked models for estimating badger sett cluster abundance using distance sampling analyses, with the associated Akaike’s information criteria (AIC and AIC_C_)

Model rank	Key model	Covariates	Number of parameters	AIC	ΔAIC	AICc	ΔAICc	GOF Chi-p
5	HR		2	875.30	18.64	875.35	18.49	0.81
		Conventional Distance Sampling						
**1**	**HR**	**SIT**	**4**	**856.66**	**0**	**856.86**	**0**	
2	HR	HAB	4	862.05	5.39	862.24	5.38	
6	HR	TER	6	880.75	24.09	881.22	24.36	
		Multiple Covariates Distance Sampling						
3	HR	SIT	3	863.25	6.59	863.34	6.48	0.66
4	HR	HAB	4	887.32	30.67	887.48	30.62	0.01
7	HR	TER	4	880.49	23.83	880.64	23.78	0.33

In bold, the top-ranked model using the hazard rate (HR) detection function

*SIT* The habitat type of the whole study site (i.e. forested or hedgerow sites), *HAB* The habitat type along the transect (i.e. forest, forest edge or hedgerows), *TER* The type of sett cluster (i.e. unoccupied, secondary, or main sett cluster)

The coefficients of variation of sett cluster density in suitable habitat *D*_C.Distance_ ranged from 22 to 40% (derived by bootstrap; n = 999), with most of the variability being due to variability in encounter rates between transects. Sett cluster density *D*_C_ calculated by multiplying *D*_C.Distance_ by the proportion of suitable habitat in each study site ranged from 1.99 (site L) to 6.42 per km^2^ (site H; Table 3).

**Table 3:** Estimates of sett cluster density from walked transect surveys of the 13 study sites in France using distance sampling

Study site	*D*_C.Distance_ (/km^2^)	*D*_C_ (/km^2^)	*D*_C_ 95% confidence interval^a^	Coefficient of variation
A	6.95	5.39	3.08–8.64	27.01
B	6.62	5.50	3.11–9.32	28.01
C	12.80	3.55	2.19–5.24	21.71
D	6.21	5.17	2.96–8.54	27.07
E	10.39	3.17	1.84–4.85	24.44
F	11.30	4.75	2.96–7.09	23.85
G	10.62	2.29	1.28–3.52	25.08
H	7.80	6.42	3.75–10.63	27.72
I	9.42	2.62	1.30–4.36	29.44
J	8.80	3.59	2.09–5.45	24.68
K	18.31	3.79	1.95–6.04	28.80
L	6.70	1.99	0.67–3.78	39.54
M	8.21	4.11	2.26–6.49	25.98

*D*_C.Distance_ and *D*_C_ correspond respectively to the density of clusters estimated in suitable habitats and corrected for the proportion of suitable habitat at each site

^a^2.5% and 97.5% quantiles of the bootstrap estimates (n = 999)

### Among-site variation in social group size and proportion of occupied sett clusters

In the 13 study sites, mean trapping effort was 515.2 ± 77.4 SD camera-trap days and 881.0 ± 300.3 SD hair-trap days per site. Based on the camera trapping survey, the mean number of adults per cluster was higher in MSCR (*ad*_MSCR_: 1.95 ± 0.43 SD, max = 3; site A) than in MSC (*ad*_MSC_*:* 1.37 ± 0.35, max = 2.25; site D) or SSC (*ad*_SSC_*:* 1.34 ± 0.41, max = 2; site C, Table 4). The average social group size, including adults and cubs, was also higher in MSCR (*badger*_MSCR_: 4.46 ± 1.01, max = 5.75; site L) than in MSC (*badger*_MSC_: 1.70 ± 0.72, max = 3.50; site B) or SSC (*badger*_SSC_: 1.68 ± 0.80, max = 3.50; site C, Table 4). At three study sites (E, I, and L; Table 4), neither camera trapping nor hair trapping allowed us to capture individuals in secondary sett clusters, so estimates of group size for these sites were calculated as the average of the estimated *ad*_SSC_ and *badger*_SSC_ for the same type of clusters (SSC) obtained in the same type of study site (i.e. forest or hedgerow sites). Similarly, we also used the average value of *ad*_MSCR_ and *badger*_MSCR_ for study site M, where no main sett clusters with reproduction were found (Table 4).

**Table 4:** Mean badger social group size for secondary (SSC) and main sett clusters (MSC and MSCR) at the 13 study sites in France, based on (i) adults using camera traps (*ad*_SSC_, *ad*_MSC_, *ad*_MSCR_) and (ii) badgers (adults and cubs) using camera traps and genetic identification (*badger*_SSC_, *badger*_MSC_, *badger*_MSCR_)

Study site	Secondary sett clusters		Main sett clusters without reproduction		Main sett clusters with reproduction	
	Mean number of adults (*ad*_SSC_)	Mean total number (adults and cubs, *badger*_SSC_)	Mean number of adults (*ad*_MSC_)	Mean total number (adults and cubs, *badger*_MSC_)	Mean number of adults (*ad*_MSCR_)	Mean total number (adults and cubs, *badger*_MSCR_)
A	1.00	1.00	1.50	2.25	3.00	5.43
B	1.00	2.00	1.75	3.50	2.29	5.71
C	2.00	3.50	1.40	1.60	2.00	4.30
D	1.60	2.00	2.25	2.50	2.20	5.20
E	1.47*	1.81*	1.13	1.13	1.75	4.50
F	1.33	1.33	1.25	1.25	2.00	4.00
G	1.50	2.00	1.00	1.00	1.67	2.67
H	1.00	1.00	1.00	1.00	1.50	4.00
I	1.47*	1.81*	1.50	1.50	2.00	5.17
J	1.00	1.00	1.25	1.75	1.40	3.40
K	2.00	2.00	1.29	1.29	1.60	3.40
L	1.47*	1.81*	1.00	1.29	2.00	5.75
M	1.00	1.00	1.56	2.11	1.80*	4.15*
Mean ± SD	1.34 ± 0.41	1.68 ± 0.80	1.37 ± 0.35	1.70 ± 0.72	1.95 ± 0.43	4.46 ± 1.01

For missing values, we considered the mean of the category for the same type of study site (forest or hedgerow sites; values indicated by *) to estimate densities (c.f. Eqs. 1 and 2)

The proportion of occupied sett clusters varied greatly among sites. For SSC, *p*_SSC_ ranged from 6% (sites E and J) to 52% (site D; mean = 18 ± 14 SD; Table 1). For MSC, *p*_MSC_ ranged from 13% (site A) to 82% (site L; mean = 33 ± 20 SD), while for MSCR, *p*_MSCR_ ranged from 0% (site L and M) to 33% (site C; mean = 13 ± 10 SD; Table 1).

### Badger population densities in France

**Fig. 1:**
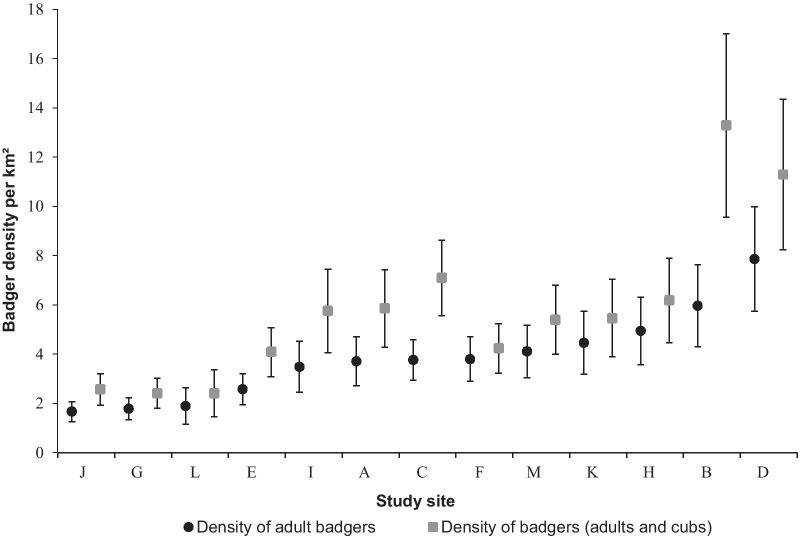
Estimates of badger densities obtained for the 13 study sites in France. Black circles indicate the minimum density in adult badgers (*D*_Ad_), considering only adults identified by camera trapping. Grey squares indicate badger density (i.e. both adults and cubs; *D*_Bad_), considering the maximum number of individuals identified by camera trapping or genetic identification surveys. Study sites were listed by increasing minimum *D*_Ad_. Error bars correspond to the standard deviations of cluster densities estimated via distance sampling

The density of adult badgers (*D*_Ad_) ranged from 1.66 (site J) to 7.86 per km^2^ (site D; Fig. 1) with an average of 3.84 ± 1.75 (SD) across study sites. Considering the total number of individuals detected per cluster, the density of adults and cubs (*D*_Bad_) ranged from 2.41 (site L) to 13.29 per km^2^ (site B; Fig. 1), with an average of 5.85 ± 3.25 across study sites.

### Determinants of spatial variation in density

**Fig. 2:**
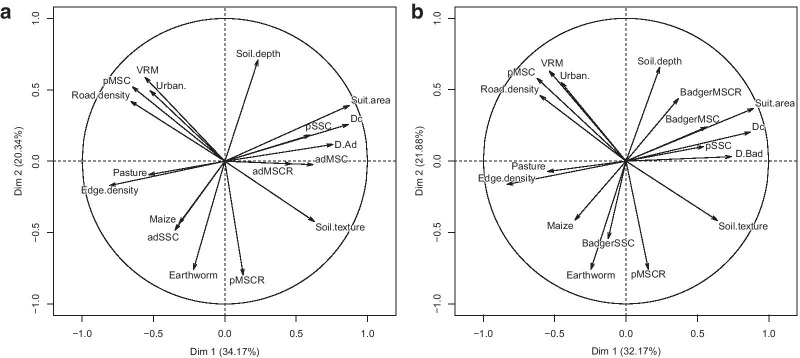
Correlation circles of the principal component analysis included the 10 environmental variables describing each study site (*Suit.area* and all variables described in Additional file 4) and **a** the adult badger density (*D*_Ad_) with the seven variables used in Eq. 1 or **b** the badger density (*D*_Bad_) with the seven variables used in Eq. 2. The sum of the eigenvalues for the first three axes of the PCAs accounted for **a** 66% and **b** 69% of the total variability

The sum of the eigenvalues for the first three axes of the principal component analysis (PCA) explaining the pattern of variability of *D*_Ad_ accounted for 66% of total variability (Fig. 2a). The first axis of this PCA accounting for more than 30% of the total variability expressed a fragmentation gradient between study sites, from fragmented landscapes (with high edge density) to more suitable habitat for badger settlement (corresponding to a greater proportion of forest, forest edge, and hedgerows; *Suit.area*). We identified seven co-variables likely to be associated with *D*_Ad_ (Fig. 2a) and for which we explicitly tested the correlation, namely: *Edge.density*, *Pasture*, *Suit.area*, *ad*_MSCR_, *ad*_MSC_, *p*_SSC_, and *D*_C_. Considering the intrinsic components, the density of adult badgers (*D*_Ad_) was positively correlated with sett cluster density *D*_C_ (rho = 0.76; p-value < 0.007 accounting for Bonferroni correction), but not significantly correlated with group sizes *ad*_MSCR_ and *ad*_MSC_ or the proportion of occupied secondary sett clusters *p*_SSC_ (all p-values > 0.007; Fig. 2a). PCA revealed that fragmented landscapes (with a high edge density) tend to support lower adult densities (rho = − 0.62; p-value = 0.027) and that suitable habitats tend to support higher badger densities (rho = 0.56; p-value = 0.05). No significant correlation was found for the presence of pasture area (rho = − 0.489; p-value = 0.093).

With regard to *D*_Bad_ (see Fig. 2b and details in Additional file 2), we only found a significant positive correlation with *Edge.density* (rho = − 0.73; p-value < 0.008 accounting for Bonferroni correction).

## Discussion

### Badger density in European countries

Densities of adult badgers (*D*_Ad_) ranged from 1.66 to 7.86 per km^2^ (average of 3.84), and *D*_Bad_ including adults and cubs ranged from 2.41 to 13.29 per km^2^ (average of 5.85) across the 13 study sites in France. Comparing badger densities across studies is inherently difficult as the methods used (distance sampling, radio-tracking, visual census, capture-recapture, group size estimation), calculations (type of sett or sett cluster accounted for or not, proportion of occupied setts accounted for or not), and estimated parameters (adult density, badger density without discrimination between adults and cubs, sett density, occupied sett density) differ among studies (see [18] for a detailed description of badger densities estimated with different methods across Europe). In the UK, populations can reach densities greater than 20 adults per km^2^ [19], but are around 9.4 individuals per km^2^ on average (ranging from 0.86 to 30.7; reviewed in [18, 20, 31, 32]. In Central Europe, densities of less than 5 individuals per km^2^ are more common [15, 33]. In Mediterranean and boreal environments, densities are lower, with badgers living in pairs or small groups at densities of less than 0.7 adults per km^2^, and in some countries densities are even lower than 0.01 individuals/km^2^ [31, 34]. Overall, our estimates were thus relatively lower than those found in the UK and concordant with global estimates from Ireland (0.72 to 11.9 adults/km^2^ depending on the region, [35]). In France, a few studies have estimated densities, most often in small areas (< 10 km^2^), with estimates ranging from 0.1 to 1.6 adults/km^2^ [36, 37], and group sizes between two and four badgers including young. Rigaux and Chanu [38] found a density of 1.9 badgers/km^2^ in a 58 km^2^ area, partly overlapping the K study site. Our estimates are clearly higher, which is mainly related to the high cluster density estimates. Cluster densities *D*_C_ ranged from 1.99 to 6.42 per km^2^ and, when taking into account the proportion of occupied main setts (ranging from 0.25 to 0.82), the densities of occupied main clusters roughly corresponded to the density of family groups, ranging from 0.76 to 2.63 per km^2^ (mean 1.72 ± 0.52 SD).

### A novel methodological approach to estimate density

Various methodological approaches have been used in previous studies to estimate badger densities; all present pros and cons. The main difference in our approach is the distance sampling method we used, which permits non-exhaustive prospection of a study area. However, we found small effective strip width in the distance sampling analysis, due to short distances between spotted setts and transects; this might have resulted in overestimated cluster densities, albeit it was comparable between study sites. The other originality and strength of our study lies in the combined use of multiple methodological approaches, namely distance sampling, camera trapping, and genetic sampling. For example, group sizes are necessarily underestimated using only camera traps as it is highly unlikely that all individuals are seen simultaneously. Here, we partially circumvented this risk by combining the use of genetic sampling, to distinguish individuals, and camera trapping to distinguish adults from cubs. Furthermore, this allowed us to fully decompose the estimation of badger density in sets of components to better understand spatial variation in population density. Our methodological approach could be applied to the long-term monitoring of a single population to better grasp temporal variation in badger density in future studies. Beyond this, for group-living and social species that live in burrows (such as meerkats *Suricata suricatta*) or do not live in burrows (such as numerous primates), composite estimates of population density over a large scale might help us better understand population dynamics, population genetics, and evolutionary ecology.

We acknowledge that our analyses may suffer from some biases, but we tried to minimise them. First, we estimated sett occupancy over a short time period (1 week) and used a standardised activity scoring system, to reduce the risk of overestimation, even though badgers regularly move between setts (especially secondary ones), and occupation cues (for example faeces, traces of digging activities) persist over a longer time period. As a result, our estimated proportion of main setts (17.3%) versus secondary and unoccupied setts (82.7%) is very similar to estimates in other populations (e.g. 22.2% for main setts and 77.8% for secondary and unoccupied setts across six studies performed in Ireland, reviewed in [20]). Another methodological limitation may be the choice of a between-setts distance of 500 m to group setts into sett clusters. The selected distance necessarily affects the number of social groups and, indeed, density estimates. Using two alternative clustering solutions (i.e. between-setts distance of 100 and 900 m), we partially confirmed this overall trend, but estimates based on these two alternative clustering solutions fell within the confidence interval of the current density estimates. We also found a strong positive correlation between badger densities calculated with the 500 m distance and the 100 m distance (rho = 0.967 and 0.918 for *D*_Ad_ and *D*_Bad_ respectively) or the 900 m distance (rho = 0.841 and 0.967 for *D*_Ad_ and *D*_Bad_ respectively; Additional file 3), supporting the rank of densities among study sites and thus PCA results.

### On the relative importance of intrinsic density components and their link with ecological variables

Decomposition of density estimation into a set of intrinsic components enables a better understanding of the spatial variation in badger population densities. Among these components, variation in sett cluster density drives the variation in adult density *D*_Ad_, while group size in main sett clusters (with and without reproduction) and the proportion of occupied secondary sett clusters are less related. In fact, group sizes are quite constant in France (see Table 4) in comparison to other European countries [19, 35]. Therefore, a better proxy for *D*_Ad_ might be sett cluster density, and researchers might focus on this parameter when seeking badger density estimates with a reduced version of our standardised protocol due to logistic constraints. PCA results have also shown that *D*_Ad_ is associated with ecological/extrinsic components. A higher proportion of suitable habitat in a study site tends to lead to greater *D*_Ad_, while more fragmented areas tend to decrease density. The major intrinsic component here that triggers spatial variation in density, i.e. sett cluster density, is thus driven by landscape fragmentation and thus the availability of suitable sett sites. Indeed, the highest *D*_Ad_ densities were found in sites dominated by large forest patches (study sites A, B, D, and H). The great importance of habitat structure for population density has also been demonstrated in other mammal species (group size of blackbuck antelope, *Antilope cervicapra*, increases with increasing habitat openness [39]) and bird species (abundance is mainly affected by habitat cover in most neotropical bird species [40]). Yet the underlying mechanisms (e.g. decreased vigilance costs, increased resource availability) often remain difficult to target without breaking down the density estimate into its structural components, as we performed here. Interestingly, the proportion of urban areas and density of roads over study sites are not related to adult badger density, but to some intrinsic components of *D*_Ad_ density. The proportion of occupied main sett clusters without reproduction (*p*_MSC_) increases in more urbanised study sites with higher road density (e.g. site L), suggesting that anthropic presence is less favourable to reproduction, whereas the proportion of main sett clusters with reproduction (*p*_MSCR_) was associated with earthworm abundance, suggesting an association between the amount of available resources and probability that a social group will reproduce efficiently. The importance of resource availability for badger settlement was already clear from an earlier study showing that habitats with a large earthworm biomass support high badger densities [41]. This result is in line with other empirical studies on social species, showing that an important component of population abundance, namely group size, is correlated with food resource availability (in caviomorph rodents, reviewed in [42]; in wolves, *Canis lupus* [43]; in blackbuck antelope, *Antilope cervicapra* [39]). High habitat quality, the availability of food resources in particular, may promote site occupancy and improve individual conditions, thereby increasing both components of individual or group fitness, i.e. reproductive success and survival. Maximum population density could thus be achieved in places where each density component is maximised, with each of these depending on different environmental factors. Whether or not these optima in different ecological factors can be reached together in some places remains unknown, but would help us better understand the limits of observable population density [44]. This also would allow us to better define the ecological requirements of a given species and better appraise on which environmental factor to focus to derive appropriate management and conservation measures.

## Conclusions

The composite estimation of badger density, in several study sites and over a large spatial scale, enabled a better understanding of their pattern of spatial variation. Densities of adult badgers (*D*_Ad_) ranged from 1.66 to 7.86 per km^2^ across the 13 study sites in France. We found that sett cluster density variation drives adult density *D*_Ad_. A higher proportion of suitable habitat tends to lead to greater *D*_Ad_, while more fragmented areas tend to decrease adult badger density. Across the geographical range of widely distributed species, habitat and environmental conditions are likely to be diverse, imposing contrasting pressures on local population abundances, as shown in various taxa (in birds [5, 7]; in 11 species of birds, mammals and reptiles [6]; in a meta-analysis [45]). Breaking down population density in order to identify which components among intrinsic and extrinsic factors trigger the variation in local density is all the more important for social species, in which population densities depend on several factors: territory size, number of social groups, and group size [43]. Identifying ecological drivers of variation in each of the key components of population abundance is a cornerstone of population ecology, including wildlife management of pest/invasive species and the conservation biology of threatened taxa.

## Methods

### Study sites

**Fig. 3:**
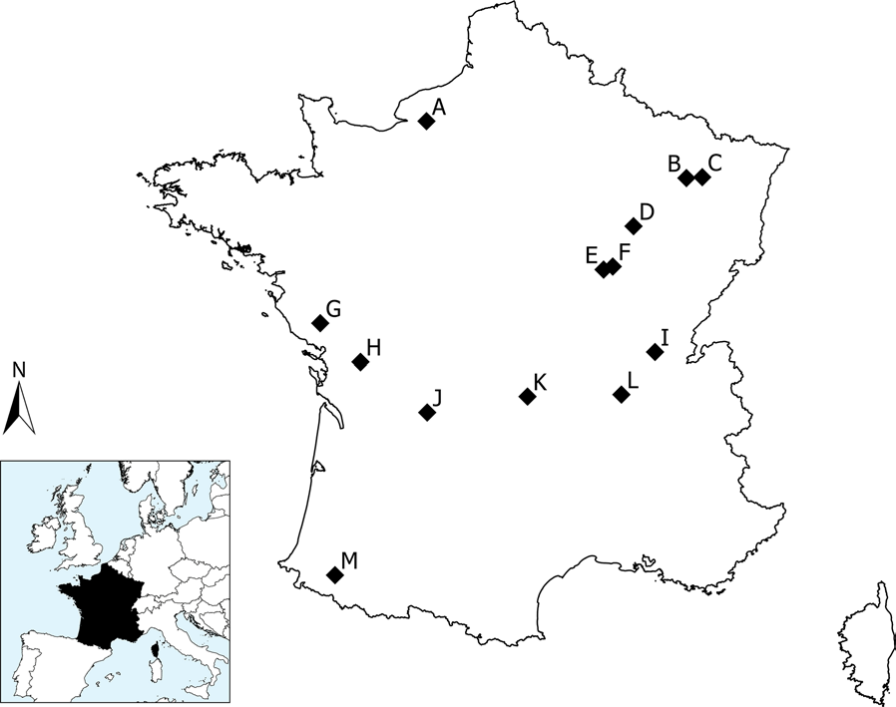
Location of the 13 study sites in France (from A to M; black diamonds). Prospected sites in 2014: G; 2016: B, D, F, H, I, and M; in 2017: A, C, E, K, and L; in 2018: J

The study was conducted at 13 sites spread throughout France (Fig. 3) between 2014 and 2018. Study sites ranged from 46.55 to 59.31 km^2^. Each site was composed of different proportions of suitable habitat (*Suit.area*) for badger settlement (i.e. forests, forest edges and hedgerows; details in Additional file 4). Two kinds of landscape dominated in the various study sites: forest in A, B, D, and H study sites (at least 70% forested areas) and hedgerows in all other study sites (less than 30% forest cover). Various other environmental variables, such as the proportion of urbanised areas, edge density, and various resource variables (proportion of maize, earthworm availability; see Additional file 4) contrasted the 13 study sites.

### Using distance sampling to estimate sett cluster density

#### Sett detection during walked transects

We used a systematic sampling method by positioning over each study site an array of equidistant theoretical points spaced 1 km apart. Transects were then placed in close vicinity to each theoretical point in suitable habitats for badger settlement (i.e. forests, forest edges and hedgerows). As sett density was expected to be very low in open areas [46], we did not sample this kind of habitat, acknowledging that it might lead to a slight underestimation of population density. Locations of transects were chosen according to the relative proportions of the three types of suitable habitat within each study site. We used detailed maps (IGN BD Topo) of the study sites on QGIS v. 2.18 [47] to plan 1- to 2-km long transects to walk (see examples of maps with theoretical points and transects in Additional file 4).

Depending on study site, 48 to 53 transects (mean = 50.92 ± 1.50 SD) were surveyed once between March and April (5 weeks), i.e. before vegetation peak, to facilitate sett detection. Realized transects were recorded using a field GPS (Garmin GPSMAP 64s). As badger setts might have numerous entrances and their surface size might vary greatly [48], we recorded the GPS coordinates of the sett centroid. Vegetation cover prevented a precise measurement of perpendicular distances between setts and transects, so we calculated perpendicular distances from tracks and sett centroids on QGIS.

#### Distinction between different sett types and sett grouping into sett clusters

There are different types of setts in badger populations: those commonly described as main setts (large and permanently occupied by all members of a social group, and used for breeding), and other setts intermittently occupied by badgers (i.e. classified as annex, subsidiary and outlier setts [49]). Classifying these intermittently occupied setts in the field requires multiple visits and follow up, so we grouped them as ‘secondary’ setts [17, 50].

Therefore, in April, after the 5-week survey to detect setts, we visited all the georeferenced setts again during a 5-day field session to assess activity (such as number of entrances or well-used badger paths, latrines, or ‘playing areas’ with denuded vegetation caused by cubs and adults chasing each other). Based on these signs of activity, we developed a standardised activity scoring system for all the study sites, and controlled for observer bias (Additional file 5). Estimated activity scores allowed us to distinguish between occupied main setts, occupied secondary setts, and unoccupied setts.

We also had to consider that the territory of a social group could encompass several setts of different types [51], forming a ‘cluster’ of setts. We thus grouped the nearest setts together and considered sett clusters rather than setts. Based on both the studies of Carter et al. ([22, 52]), and on the distance distribution between the detected setts in all study sites (Additional file 4), we considered a between-setts centroid distance of 500 m. This distance seemed a reliable distance to group setts into clusters while excluding setts from neighbouring social groups. As a result, clusters could be composed of one or several detected setts of different types (see maps in Additional file 4). It is also well-known that not all females and social groups produce cubs every year [53]. Hence, we considered four categories: sett clusters with (i) at least one occupied main sett with reproduction as main sett clusters with reproduction (MSCR), (ii) at least one occupied main sett without reproduction as main sett clusters (MSC), (iii) only secondary setts with at least one occupied as secondary sett clusters (SSC), and (iv) only unoccupied setts. Those categories were then used for calculations of sett clusters density and badger group sizes. We also determined per study site the proportions of the occupied main sett clusters with reproduction (*p*_MSCR_), occupied main sett clusters without reproduction (*p*_MSC_), and occupied secondary sett clusters (*p*_SSC_; Table 1).

#### Distance sampling analysis

We used distance sampling methodology [23, 24], in which the probability of detection is modelled from the frequency distribution of the perpendicular distances of detected clusters from the transect line. We calculated the perpendicular distance between the first detected sett centroid in the cluster and the walked transect using QGIS v 2.18 [47]. Following the recommendations of Buckland et al. [23], we right truncated the data by removing 3.3% of observations that were more than 55 m from the transect. We tested five a priori robust models to fit the probability of detection: a uniform key function with either cosine or polynomial series expansion, a half-normal with either cosine or Hermine polynomial series expansion and a HR key function with cosine series expansion. We assessed their goodness of fit visually and through Chi-squared tests. In modelling the detection function, we expected that several covariates could a priori affect detection probability: (i) HAB, the habitat type along the transect (i.e. forest, forest edge or hedgerow), (ii) SIT, the habitat type of the whole study site (i.e. forested or hedgerow sites), and (iii) TER, the type of sett clusters. We used multiple-covariates distance sampling (MCDS, [54, 55]) and tested post-stratification with the conventional distance sampling (CDS) method. MCDS tested whether the scale parameter (and hence the shape of the detection function) varied between levels of a covariate, whereas CDS tested for different detection functions (shape and origin). We retained the best function model ranked by the Akaike information criteron (AIC).

To estimate the density of sett clusters in suitable habitat by study site (*D*_C.Distance_; Table 3), we used stratum-specific detection probabilities. The effective strip width was based on the best model, and encounter rates were calculated for each study site. We derived confidence intervals from a non-parametric bootstrap method, resampling transects within each study site to estimate bootstrap variances of the encounter rates (with n = 999). We then derived the density of sett clusters in each site (*D*_C_, Table 3) by multiplying each *D*_C.Distance_ by the proportion of suitable habitat in the corresponding study site. All analyses were conducted in DISTANCE software 7.3 [55]. A DISTANCE project containing the data and analyses is available from the authors.

### Determination of badger group size per sett cluster

In order to estimate social group size of badgers per cluster type (i.e. MSCR, MSC, and SSC), we used two non-invasive methods, i.e. camera trapping and hair traps for genetic identification. Due to material and human limitations, we were unable to follow all occupied setts detected in the study. We selected on average 18 occupied setts (± 4 SD [min = 9; max = 27]) from each study site (with approximately half of the main setts and half of the secondary setts). Between April and June, we deployed camera traps on these selected setts for 2 weeks and hair traps for 2 another weeks, alternating the methods between setts to avoid bias. Due to the small number of occupied setts detected in seven study sites, we had to include setts found outside the walked transects protocol, that were known to local people (see Table 1 for the total number of setts used per site). Depending on the study site, the number of occupied setts and their spatial localisation, several setts of the same cluster were surveyed. Badger group size per sett cluster corresponds to the maximum number of animals detected among all setts in the cluster.

#### Camera trapping survey

We monitored setts using one to five infrared cameras, depending on the sett type (mean = 2.41 ± 0.90 for main setts; and 1.47 ± 0.74 for secondary setts). Camera traps were tied to trees about 5 m from active entrances, badger paths, and preferably in any places with recent signs of activity and gathering (for example ‘grooming and playing areas’), in order to detect all adults and cubs present in the sett. We checked for battery depletion and proper functioning of each camera trap twice during the 2-week survey (after 2 and 8 days in the field).

The camera trap method allowed us to distinguish adults and cubs based on body size, and thus estimate the mean number of adults per cluster type (*ad*_MSCR_, *ad*_MSC_, and *ad*_SSC_; Table 4). For a given sett cluster, we took the maximum number of adults seen together on both pictures and videos. These social group sizes were minima, as we did not have the absolute certainty that all individuals living in a sett cluster were camera-trapped.

#### Hair trapping survey for genetic identification

We sampled badger hairs using hair traps, i.e. barbed wire (thickness of 1.7 mm, with barbs spread every 10 cm) suspended over visible badger paths, approximately 20 cm above ground level, to subsequently identify individuals genetically. Hair samples were genotyped at 24 microsatellite markers and one sex marker (full protocol described in [52]). We identified a total of 284 badgers (145 males and 139 females). Genetic identification did not allow us to distinguish between adults and young individuals. As for the camera trap estimates, hair trap estimates were minima, and we selected the maximum of both estimates, i.e. from camera traps and genetic identification, as the badger group size per cluster type (*badger*_MSCR_, *badger*_MSC_ and *badger*_SSC_; Table 4). The study site G conducted in 2014 was a pilot study site, and the hair trapping survey could not be put in place, so the mean badger group size was estimated only from camera-trap results at this site.

### Badger density estimates

For each study site, we estimated adult density *D*_Ad_ and badger density *D*_Bad_ (i.e. adults and cubs) per square kilometre as composites of their different components, as follows:

1 $${D_{\text{Ad}}} = {D_\text{C}} \times \left( {{p_{\text{SSC}}} \times {ad_{\text{SSC}}} + {p_{\text{MSC}}} \times {ad_{\text{MSC}}} + {p_{\text{MSCR}}} \times {ad_{\text{MSCR}}}} \right)$$

and

2 $${D_{\text{Bad}}}={D_{\text{C}}} \times ({p_\text{SSC}} \times{badger_\text{SSC}}+{p_\text{MSC}} \times {badger_\text{MSC}}+{p_\text{MSCR}} \times {badger_\text{MSCR}})$$

where *D*_C_ is the density of sett clusters in km^2^ estimated by distance sampling and corrected for the proportion of suitable habitat in each site; *p*_SSC_, *p*_MSC_, and *p*_MSCR_ are the proportions of occupied SSC, MSC, and MSCR among all sett clusters; *ad*_SSC_, *ad*_MSC_, and *ad*_MSCR_ are the mean number of adults per SSC, MSC, and MSCR surveyed in the corresponding study site; and *badger*_SSC_, *badger*_MSC_, and *badger*_MSCR_ are the mean social group size in badgers (adults and cubs) per SSC, MSC, and MSCR, as above.

### Determinants of spatial variation in density

In order to identify which ecological factors influenced spatial variation in density, we performed a PCA using the *FactomineR* package [56] operating in R software [57]. We included in the PCA adult density (*D*_Ad_), each intrinsic component used in Eq. 1 (n = 7), and extrinsic factors linked with local environmental characteristics (n = 10). The environmental variables included were *Suit.area*, *Edge density* (m/ha), *VRM* (°), *Soil texture* and *depth* (indices), *Earthworm* abundance (ind./m^2^), percentage of *Pasture*, *Maize*, *Urbanised area* in the study site, and *Road density* (km/km^2^), defined in Additional file 4. PCA allowed us to identify a reduced set of variables, both intrinsic and extrinsic, likely to be associated with *D*_Ad_; we further explicitly tested for the association using non-parametric Spearman’s rank correlation tests (R package *pspearman*, [58]). p-values were adjusted using the Bonferroni procedure for multiple comparisons [59] on the reduced set of variables. We applied the same procedure on badger density, adults and cubs, by substituting *D*_Ad_ by *D*_Bad_ and using the intrinsic components in Eq. 2. Coefficients of variation (CV) of each variable included in the PCAs are available in Additional file 2.

## Supplementary Information

**Additional file 1.** Detection functions for badger sett clusters estimated using distance sampling methodology.

**Additional file 2.** Coefficients of variation of each variable included in the PCAs explaining the pattern of variability of badger density.

**Additional file 3.** Estimations of badger density over the 13 study sites using two alternative clustering solutions.

**Additional file 4.** Description and geographic maps of the 13 study sites surveyed in France.

**Additional file 5.** Standardized scoring system used in order to distinguish badger main setts, occupied secondary setts, and unoccupied setts across all study sites.

## Data Availability

The datasets used and/or analysed during the current study are available from the corresponding author on reasonable request.

## Abbreviations

*ad*_SSC_: Mean number of adult badgers in secondary sett cluster
*ad*_MSC_: Mean number of adult badgers in main sett cluster without reproduction
*ad*_MSCR_: Mean number of adult badgers in main sett cluster with reproduction
*badger*_SSC_: Mean number of adult and cubs in secondary sett cluster
*badger*_MSC_: Mean number of adult and cubs in main sett cluster without reproduction
*badger*_MSCR_: Mean number of adult and cubs in main sett cluster with reproduction
CDS: Conventional distance sampling
*D*_Ad_: Adult badger density
*D*_Bad_: Adult and cubs density
*D*_C.Distance_: Cluster density estimated in suitable habitats of each site
*D*_C_: Cluster density in each site, i.e. *D*_C.Distance_ × [proportion of suitable habitat in the site]
HAB: Habitat type along the transect (i.e. forest, forest edge or hedgerows)
MCDS: Multiple covariates distance sampling
MSC: Main sett cluster without reproduction
MSCR: Main sett cluster with reproduction
PCA: Principal component analysis
p_SSC_: Proportion of occupied secondary sett clusters
p_MSC_: Proportion of occupied main sett clusters without reproduction
p_MSCR_: Proportion of occupied main sett clusters with reproduction
SIT: Habitat type of the whole study site (i.e. forested or hedgerow sites)
SSC: Secondary sett cluster
TER: Type of sett cluster (i.e. unoccupied, secondary or main sett cluster)
