# Supplementary material for: Breaking down population density into different components to better understand its spatial variation
Source: BMC Ecol Evol. 2021 May 11;21:82. doi: 10.1186/s12862-021-01809-6 (PMC8111954; doi:10.1186/s12862-021-01809-6)
Supplement: Supplementary file 1 — Additional file 1. Detection functions for badger sett clusters estimated using distance sampling methodology. [file 12862_2021_1809_MOESM1_ESM.docx]

**Additional file 1**

**Fig. S1.1.** Fitted hazard-rate detection functions for badger sett clusters by perpendicular distance from the transect line for (A) hedgerow sites (n = 9) and (B) forest sites (n = 4).

1. Hedgerow sites (n = 169, p-value = 0.50, chi2 = 3.35, df = 4)

**
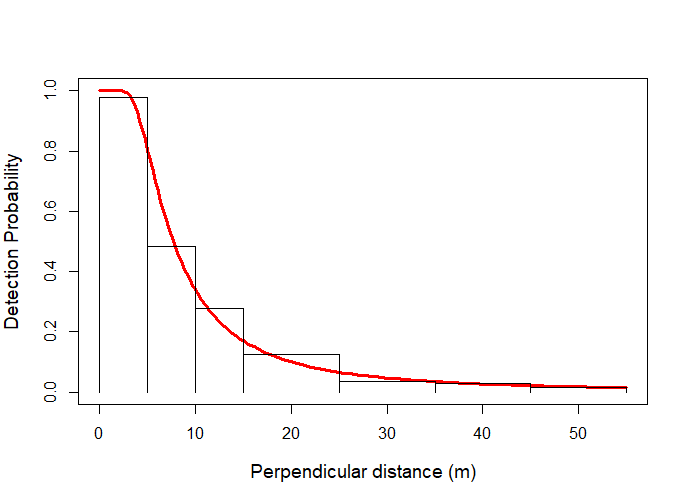
**

1. Forest sites (n = 95, p-value = 0.99, chi2 = 0.18, df = 4)

**
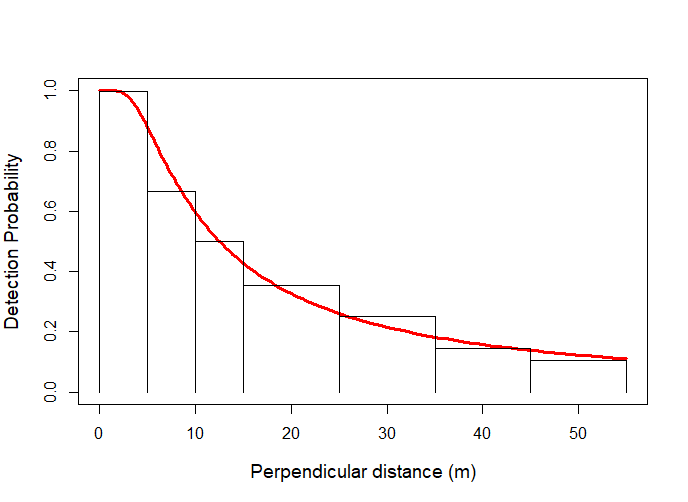
**
