# Supplementary material for: Breaking down population density into different components to better understand its spatial variation
Source: BMC Ecol Evol. 2021 May 11;21:82. doi: 10.1186/s12862-021-01809-6 (PMC8111954; doi:10.1186/s12862-021-01809-6)
Supplement: Supplementary file 2 — Additional file 2. Coefficients of variation of each variable included in the PCAs explaining the pattern of variability of badger density. [file 12862_2021_1809_MOESM2_ESM.docx]

**Additional file 2**

The sum of the eigenvalues for the first three axes of the PCA explaining the pattern of variability of ***D_Ad_*** accounted for 66% of the total variability (see Fig. 2a in the main paper). The first axis of this PCA accounting for more than 30% of the total. We identified 7 co-variables likely associated with *D_Ad_* and for which we explicitly test the correlation (see Table S2.1 below), namely: *Edge.density*, *Pasture*, *Suit.area*, *ad_MSCR_*, *ad_MSC_*, *p_SSC_* and *D_C_*.

**Table S2.1.** Correlation matrice between the adult badger density (*D_Ad_*) obtained for each study site and the intrinsic variables used in the Equation 1 (n = 7, in black) and the environmental variables describing each area (n = 10, in green), using the spearman method on the 7 co-variables likely associated with *D_Ad_*.

|  | **Coefficient of variation** | **rho** | **p-value** |
| --- | --- | --- | --- |
| *D_Ad_* | 0.46 |  |  |
| *Dc* | 0.34 | 0.764 | 0.003* |
| *p_SSC_* | 0.77 | 0.680 | 0.013 |
| *Edge density* | 0.85 | -0.621 | 0.027 |
| *ad_MSC_* | 0.26 | 0.571 | 0.045 |
| *Suit.area* | 0.53 | 0.560 | 0.050 |
| *Pasture* | 1.09 | -0.489 | 0.093 |
| *ad_MSCR_* | 0.21 | 0.326 | 0.277 |
| *ad_SSC_* | 0.26 |  |  |
| *p_MSC_* | 0.61 |  |  |
| *p_MSCR_* | 0.74 |  |  |
| *Soil texture* | 0.30 |  |  |
| *Road density* | 0.36 |  |  |
| *Soil depth* | 0.43 |  |  |
| *VRM* | 0.62 |  |  |
| *Earthworm* | 0.65 |  |  |
| *Maize* | 1.39 |  |  |
| *Urban.* | 1.58 |  |  |

* significant p-value using Bonferoni correction (p-value < 0.007)

The second PCA was carried out with the badger density ***D_Bad_*** and using the intrinsic components in Equation 2 of the main paper. The sum of the eigenvalues for the first three axes of the PCA accounted for 69% of the total variability (see Fig. 2b in the main paper). The first axis also expressed a fragmentation gradient between study sites and accounting for more than 30% of the total variability. We identified 6 co-variables likely associated with *D_Bad_*, and for which we explicitly test the correlation (see Table S2.2 below), namely: *Edge.density*, *Pasture*, *Suit.area*, *badger_MSC_*, *p_SSC_* and *D_C_*.

**Table S2.2.** Correlation matrice between the badger density (*D_Bad_*) obtained for each study site and the intrinsic variables used in the Equation 1 (n = 7, in black) with the environmental variables describing each area (n = 10, in green), using the spearman method on the 6 co-variables likely associated with *D_Bad_*.

|  | **Coefficient of variation** | **rho** | **p-value** |
| --- | --- | --- | --- |
| *D_Bad_* | 0.57 |  |  |
| *Edge density* | 0.85 | -0.725 | 0.007* |
| *Dc* | 0.34 | 0.703 | 0.009 |
| *Pasture* | 1.09 | -0.577 | 0.043 |
| *badger_MSC_* | 0.42 | 0.551 | 0.055 |
| *Suit.area* | 0.53 | 0.505 | 0.081 |
| *p_SSC_* | 0.77 | 0.393 | 0.185 |
| *badger_MSCR_* | 0.22 |  |  |
| *badger_SSC_* | 0.40 |  |  |
| *p_MSC_* | 0.61 |  |  |
| *p_MSCR_* | 0.74 |  |  |
| *Soil texture* | 0.30 |  |  |
| *Road density* | 0.36 |  |  |
| *Soil depth* | 0.43 |  |  |
| *VRM* | 0.62 |  |  |
| *Earthworm* | 0.65 |  |  |
| *Maize* | 1.39 |  |  |
| *Urban.* | 1.58 |  |  |

* significant p-value using Bonferoni correction (p-value < 0.008)
