# Supplementary material for: Breaking down population density into different components to better understand its spatial variation
Source: BMC Ecol Evol. 2021 May 11;21:82. doi: 10.1186/s12862-021-01809-6 (PMC8111954; doi:10.1186/s12862-021-01809-6)
Supplement: Supplementary file 3 — Additional file 3. Estimations of badger density over the 13 study sites using two alternative clustering solutions. [file 12862_2021_1809_MOESM3_ESM.docx]

**Additional file 3**

In this supplementary, we estimated both the adult and badger (adults and cubs) density per km² over the 13 study sites using two alternative clustering solutions (i.e. between-setts distance of 100 m and 900 m; instead of 500 m in the main paper).

Firstly, we present the distance sampling results obtained for these new distance classes, and the selected detection functions on the histogram of the observed perpendicular distance data (Table S3.1; S3.2 and Fig. S3.1; S3.2). Secondly, we calculated the density estimates for each distance classes (Fig. S3.3) and the percentage of variation between classes 100 m – 500 m and 900 m – 500 m for each study site (Table S3.3; S3.4). The correlation between the different densities (*D_Ad_* and *D_Bad_*) obtained with the different clustering solutions were tested over all study sites using the non-parametric Spearman’s rank correlation test (R package *pspearman*, [1]; Table S3.5).

*Distance sampling results using a between-sett centroid distance of 100 m to group setts into clusters:*

**Table S3.1.** Parameters estimates of the 7 top ranked models for estimating badger sett cluster abundance using distance sampling analyses, with the associated Akaike’s Information Criteria (AIC and AIC_C_). The top ranked models used the hazard rate (HR) detection fonction.

| **Model rank** | **Key model** | **Covariates** | **Number of parameters** | **AIC** | **ΔAIC** | **AICc** | **ΔAICc** | **GOF Chi-p** |
| --- | --- | --- | --- | --- | --- | --- | --- | --- |
| 5 | HR |  | 2 | 966.93 | 48.71 | 966.97 | 48.61 | 0.72 |
|  |  | ***CDS*** |  |  |  |  |  |  |
| **1** | **HR** | **SIT** | **4** | **918.23** | **0** | **918.36** | **0** |  |
| 2 | HR | HAB | 4 | 932.93 | 14.70 | 933.06 | 14.70 |  |
| 6 | HR | TER | 8 | 972.87 | 54.64 | 973.67 | 55.32 |  |
|  |  | ***MCDS*** |  |  |  |  |  |  |
| 3 | HR | SIT | 3 | 927.80 | 9.58 | 927.87 | 9.51 | 0.53 |
| 4 | HR | HAB | 4 | 937.98 | 19.75 | 938.08 | 19.73 | 0.09 |
| 7 | HR | TER | 8 | 973.88 | 55.66 | 974.26 | 55.90 |  |

**CDS** = Conventional Distance Sampling.

**MCDS** = Multiple Covariates Distance Sampling.

**SIT** = The habitat type of the whole study site (i.e. forested or hedgerow sites).

**HAB** = The habitat type along the transect (i.e. forest, forest edge or hedgerows).

**TER** = The type of sett cluster (i.e. unoccupied, secondary or main sett cluster).

**Fig. S3.1.** Fitted hazard-rate detection functions for badger sett clusters by perpendicular distance from the transect line for (A) hedgerow sites (n = 9) and (B) forest sites (n = 4).

1. Hedgerow sites (n = 229, p-value = 0.86, chi2 = 0.77, df = 3)

**
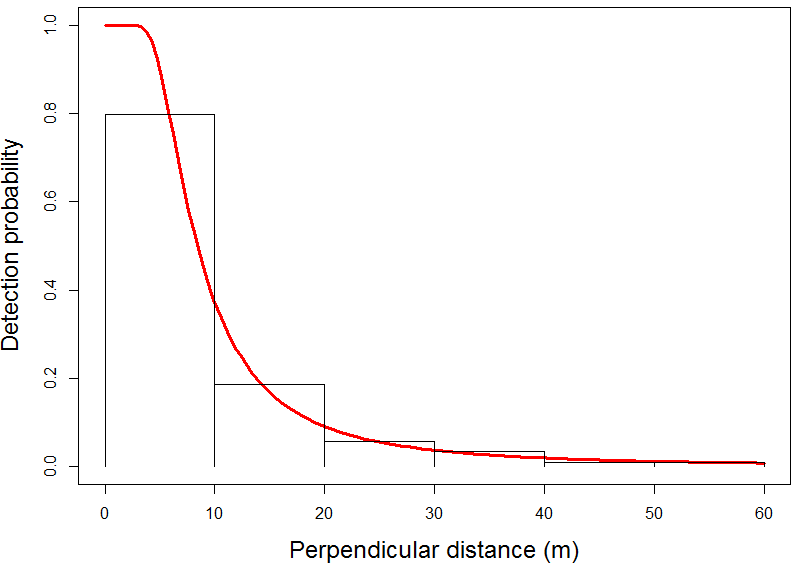
**

1. Forest sites (n = 165, p-value = 0.44, chi2 = 2.70, df = 3)

**
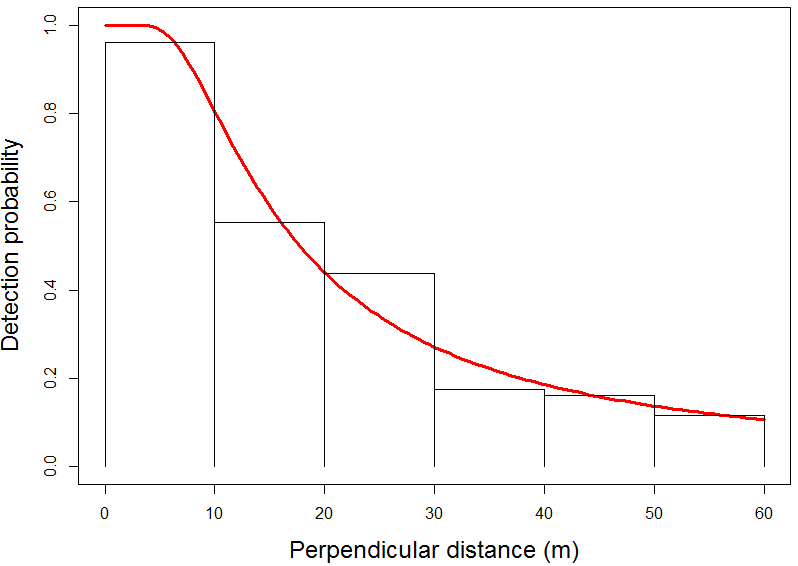
**

*Distance sampling results using a between-sett centroid distance of 900 m to group setts into clusters:*

**Table S3.2.** Parameters estimates of the 7 top ranked models for estimating badger sett cluster abundance using distance sampling analyses, with the associated Akaike’s Information Criteria (AIC and AIC_C_). The top ranked models used the hazard rate (HR) detection fonction.

| **Model rank** | **Key model** | **Covariates** | **Number of parameters** | **AIC** | **ΔAIC** | **AICc** | **ΔAICc** | **GOF Chi-p** |
| --- | --- | --- | --- | --- | --- | --- | --- | --- |
| 5 | HR |  | 2 | 564.85 | 8.56 | 564.92 | 8.20 | 0.44 |
|  |  | ***CDS*** |  |  |  |  |  |  |
| **1** | **HR** | **SIT** | **4** | **323.15** | **0** | **323.47** | **0** |  |
| 2 | HR | HAB | 4 | 328.85 | 5.70 | 329.14 | 5.67 |  |
| 6 | HR | TER | 8 | 342.13 | 18.98 | 343.53 | 20.06 |  |
|  |  | ***MCDS*** |  |  |  |  |  |  |
| 3 | HR | SIT | 3 | 339.30 | 16.15 | 339.43 | 15.96 | 0 |
| 4 | HR | HAB | 3 | 339.36 | 16.21 | 339.50 | 16.02 | 0 |
| 7 | HR | TER | 5 | 343.36 | 20.21 | 343.71 | 20.23 |  |

**CDS** = Conventional Distance Sampling.

**MCDS** = Multiple Covariates Distance Sampling.

**SIT** = The habitat type of the whole study site (i.e. forested or hedgerow sites).

**HAB** = The habitat type along the transect (i.e. forest, forest edge or hedgerows).

**TER** = The type of sett cluster (i.e. unoccupied, secondary or main sett cluster).

**Fig. S3.2.** Fitted hazard-rate detection functions for badger sett clusters by perpendicular distance from the transect line for (A) hedgerow sites (n = 9) and (B) forest sites (n = 4).

1. Hedgerow sites (n = 123, p-value = 0.23, chi2 = 4.26, df = 3)

**
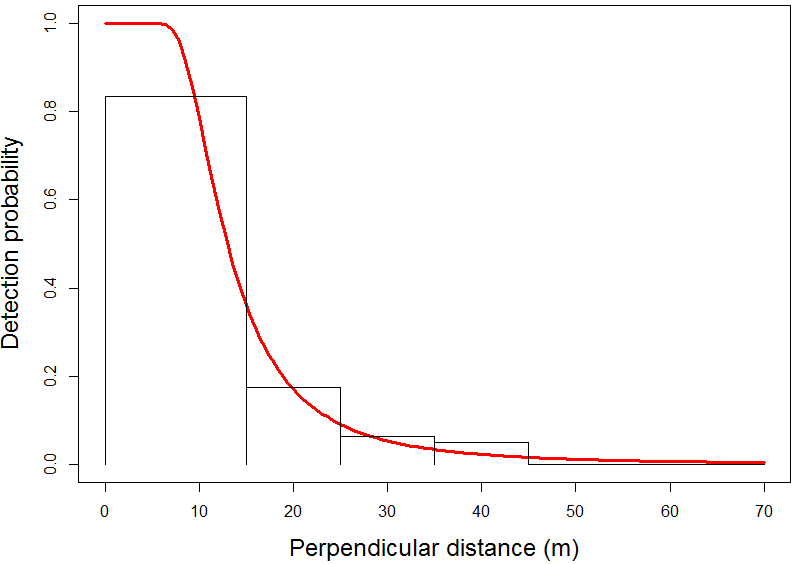
**

1. Forest sites (n = 57, p-value = 0.35, chi2 = 3.30, df = 3)

**
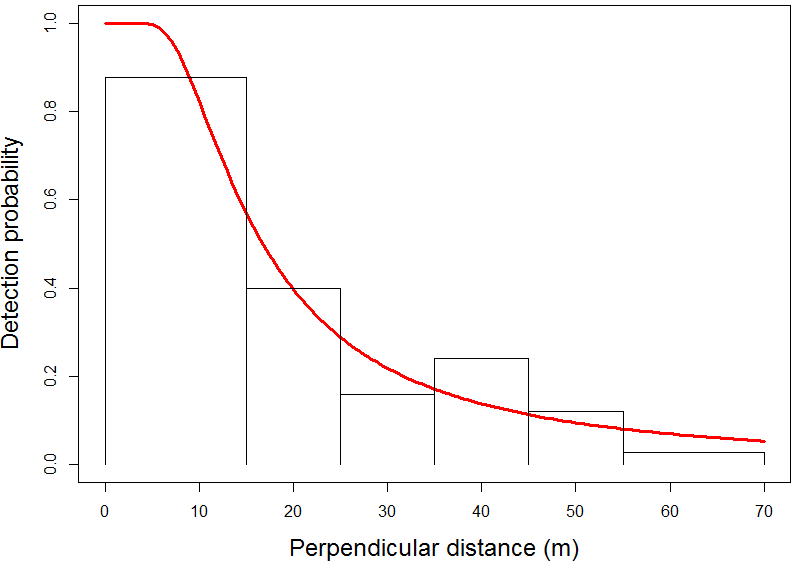
**

**Fig. S3.3.** Estimates of badger densities per km² obtained for the thirteen study sites in France, considering the three different sizes of clusters, in (A) adult *D_Ad_* and (B) badgers -both adults and cubs- *D_Bad_*.

**(A)**

**(B)**

**Table S3.3.** Density in adult badger per km² (*D_Ad_*) obtained for the thirteen study sites in France, considering different sett clustering solutions (i.e. 100; 500 and 900 m), with the percentage of variation between classes.

|  | **Density in adult badger (*D_Ad_*)** | | | **% Variation** | |
| --- | --- | --- | --- | --- | --- |
| **Study site** | **100 m** | **500 m** | **900 m** | **100 - 500 m** | **900 - 500 m** |
| **A** | 3.31 | 3.71 | 3.37 | -10.78 | -8.98 |
| **B** | 6.53 | 5.96 | 4.44 | 9.55 | -25.54 |
| **C** | 3.48 | 3.76 | 2.29 | -7.47 | -39.15 |
| **D** | 10.49 | 7.86 | 4.70 | 33.55 | -40.22 |
| **E** | 2.35 | 2.57 | 1.83 | -8.70 | -28.59 |
| **F** | 4.01 | 3.79 | 2.34 | 5.69 | -38.41 |
| **G** | 1.79 | 1.78 | 1.18 | 0.53 | -33.83 |
| **H** | 4.96 | 4.94 | 4.09 | 0.34 | -17.18 |
| **I** | 3.52 | 3.49 | 2.71 | 1.05 | -22.25 |
| **J** | 1.06 | 1.66 | 1.37 | -36.52 | -17.39 |
| **K** | 3.75 | 4.46 | 2.27 | -15.79 | -48.97 |
| **L** | 1.88 | 1.89 | 1.30 | -0.31 | -31.25 |
| **M** | 4.79 | 4.11 | 2.87 | 16.61 | -30.19 |
| ***Mean ± SD*** | **3.99 ± 2.48** | **3.84 ± 1.75** | **2.67 ± 1.18** | **-0.94 ± 16.70** | **-29.38 ± 11.10** |
| ***Min*** | 1.06 | 1.66 | 1.18 | -36.52 | -48.97 |
| ***Max*** | 10.49 | 7.86 | 4.70 | 33.55 | -8.98 |

**Table S3.4.** Density in badgers per km² (*D_Bad_*) obtained for the thirteen study sites in France, considering different sett clustering solutions (i.e. 100; 500 and 900 m), with the percentage of variation between classes.

|  | **Density in badgers (*D_Bad_*)** | | | **% Variation** | |
| --- | --- | --- | --- | --- | --- |
| **Study site** | **100 m** | **500 m** | **900 m** | **100 - 500 m** | **900 - 500 m** |
| **A** | 4.58 | 5.85 | 5.32 | -21.74 | -8.98 |
| **B** | 13.69 | 13.29 | 9.88 | 2.98 | -25.66 |
| **C** | 6.30 | 7.09 | 4.43 | -11.14 | -37.58 |
| **D** | 13.88 | 11.29 | 7.98 | 22.92 | -29.32 |
| **E** | 3.82 | 4.08 | 2.92 | -6.44 | -28.50 |
| **F** | 4.43 | 4.22 | 2.65 | 4.93 | -37.28 |
| **G** | 2.43 | 2.42 | 1.60 | 0.53 | -33.83 |
| **H** | 5.95 | 6.18 | 5.37 | -3.67 | -13.04 |
| **I** | 5.18 | 5.75 | 4.35 | -10.04 | -24.32 |
| **J** | 1.39 | 2.56 | 1.86 | -45.78 | -27.45 |
| **K** | 4.51 | 5.46 | 3.03 | -17.42 | -44.48 |
| **L** | 2.39 | 2.41 | 1.66 | -1.08 | -31.28 |
| **M** | 6.24 | 5.39 | 3.90 | 15.68 | -27.66 |
| ***Mean ± SD*** | **5.75 ± 3.87** | **5.85 ± 3.25** | **4.23 ± 2.47** | **-5.41 ± 17.27** | **-28.41 ± 9.57** |
| ***Min*** | 1.39 | 2.41 | 1.60 | -45.78 | -44.48 |
| ***Max*** | 13.88 | 13.29 | 9.88 | 22.92 | -8.98 |

**Table S3.5.** Correlation matrice between the different densities (*D_Ad_* and *D_Bad_*) obtained with the 500 m distance class clustering, and the corresponding densities obtained with the 100 and 900 m clustering solutions, over all study sites using the spearman method [1].

|  | **Coefficient of variation** | **rho** |
| --- | --- | --- |
| ***D_Ad_ 500 m*** | 0.46 |  |
| *100 m* | 0.61 | 0.967 |
| *900 m* | 0.44 | 0.841 |
|  |  |  |
| ***D_Bad_ 500 m*** | 0.56 |  |
| *100 m* | 0.67 | 0.918 |
| *900 m* | 0.59 | 0.967 |

**References**

1. Savicky P. pspearman: sprearman’s rank correlation test. R package version 0.3.0. 2014. http://cran.r-project.org/package=pspearman/
