# Supplementary material for: Breaking down population density into different components to better understand its spatial variation
Source: BMC Ecol Evol. 2021 May 11;21:82. doi: 10.1186/s12862-021-01809-6 (PMC8111954; doi:10.1186/s12862-021-01809-6)
Supplement: Supplementary file 4 — Additional file 4. Description and geographic maps of the 13 study sites surveyed in France. [file 12862_2021_1809_MOESM4_ESM.docx]

**Additional file 4**

**Table S4.1.** Description of the 13 study sites (from A to M) surveyed in France between 2014 and 2018, with the corresponding number of prospected transects. Suitable habitat (*Suit.area*) for badger settlement corresponds to the proportion of forest, forest edge and hedgerow in the whole study site.

| **Study site** | **Year** | **Study site area (km²)** | **Forest (km²)** | **Forest edge (km²)** | **Hedgerow (km²)** | **Open area (km²)** | ***Suit.area* (%)** | **Number of transects in the survey** |
| --- | --- | --- | --- | --- | --- | --- | --- | --- |
| **A** | 2017 | 53.11 | 37.19 | 5.46 | 0.45 | 10.01 | 81.16 | 51 |
| **B** | 2016 | 49.65 | 35.43 | 5.41 | 0.35 | 8.46 | 82.96 | 51 |
| **C** | 2017 | 54.26 | 7.93 | 6.25 | 0.87 | 39.20 | 27.75 | 50 |
| **D** | 2016 | 49.52 | 36.98 | 4.18 | 0.11 | 8.25 | 83.33 | 50 |
| **E** | 2017 | 56.36 | 10.66 | 5.05 | 1.48 | 39.16 | 30.51 | 52 |
| **F** | 2016 | 48.92 | 10.01 | 9.31 | 1.23 | 28.37 | 42.01 | 53 |
| **G** | 2014 | 59.31 | 4.77 | 4.39 | 3.65 | 46.50 | 21.60 | 52 |
| **H** | 2016 | 49.49 | 37.07 | 3.58 | 0.16 | 8.68 | 82.46 | 50 |
| **I** | 2016 | 46.55 | 6.81 | 5.90 | 1.06 | 32.77 | 29.61 | 49 |
| **J** | 2018 | 53.36 | 7.76 | 12.97 | 1.08 | 31.55 | 40.88 | 51 |
| **K** | 2017 | 47.72 | 0.62 | 5.61 | 3.66 | 37.84 | 20.71 | 53 |
| **L** | 2017 | 57.67 | 7.02 | 8.92 | 2.00 | 39.73 | 31.09 | 48 |
| **M** | 2016 | 49.88 | 13.98 | 9.58 | 1.38 | 24.93 | 50.02 | 52 |

**Table S4.2.** Environmental variables calculated for each of the 13 study sites (from A to M) surveyed in France. VRM corresponds to a topographical ruggedness measure. Increasing values of soil texture (from 0 to 5) and depth (from 1 to 4) corresponds to finer soil structure and less deep soil respectively. We also provide the density of earthworms (per m²) and the road density (per km²), as well as the proportion of pasture, maize, and urban area in each study site.

| **Study site** | **Edge density (m/ha)** | **VRM (°)** | **Soil texture (index)** | | **Soil depth (index)** | **Earthworm (ind./m²)** | **Pasture (%)** | **Maize (%)** | **Urban. (%)** | **Road density (km/km²)** |
| --- | --- | --- | --- | --- | --- | --- | --- | --- | --- | --- |
| **A** | 57.60 | 3.70E-04 | 3.04 | 1.22 | | 52.45 | 4.77 | 1.35 | 0.60 | 2.27 |
| **B** | 61.63 | 8.15E-04 | 3.05 | 1.64 | | 45.59 | 2.25 | 0.00 | 9.15 | 3.81 |
| **C** | 252.16 | 3.11E-04 | 3.25 | 1.00 | | 136.19 | 16.28 | 8.36 | 3.90 | 2.21 |
| **D** | 32.03 | 3.13E-04 | 2.70 | 2.63 | | 39.99 | 1.89 | 0.27 | 0.03 | 1.54 |
| **E** | 279.99 | 3.55E-04 | 3.56 | 1.00 | | 31.36 | 43.86 | 0.60 | 1.48 | 1.86 |
| **F** | 284.34 | 7.88E-04 | 2.87 | 2.48 | | 47.67 | 29.79 | 0.62 | 0.10 | 1.83 |
| **G** | 629.14 | 3.29E-04 | 2.00 | 1.00 | | 161.27 | 7.86 | 14.01 | 2.48 | 3.33 |
| **H** | 29.93 | 4.96E-05 | 2.70 | 3.20 | | 53.70 | 0.00 | 0.78 | 0.00 | 2.68 |
| **I** | 292.51 | 3.86E-04 | 2.21 | 1.64 | | 127.94 | 18.41 | 15.46 | 1.95 | 3.04 |
| **J** | 315.34 | 8.46E-04 | 1.12 | 1.83 | | 29.69 | 24.55 | 1.29 | 0.55 | 2.60 |
| **K** | 878.19 | 9.57E-04 | 1.89 | 1.55 | | 44.91 | 74.96 | 0.00 | 3.87 | 3.95 |
| **L** | 433.59 | 1.27E-03 | 1.33 | 2.85 | | 38.58 | 18.21 | 2.11 | 21.41 | 5.23 |
| **M** | 206.78 | 1.16E-03 | 2.07 | 1.19 | | 67.14 | 7.53 | 18.60 | 2.90 | 2.72 |

**Edge density (m/ha)** = Sum of the lengths of all edge segments (in meters), divided by total vegetation area (i.e. m²) multiplied by 10,000 (IGN 2015^a^).

**VRM** **(°)** = Mean Vector Ruggedness Measure (VRM) of terrain (IGN 2011^b^).

**Soil texture (index)**= Mean index of dominant surface textural class derived from clay, silt and sand topsoil maps (measured in 5 categories: from 0 = coarse to 5 = fine; BDGSF 1998^c^).

**Soil depth (index)**= Mean index of depth class of an obstacle to roots (measured in 4 categories: 1 = no obstacle to roots between 0 and 80 cm, 2 = obstacle to roots between 60 and 80 cm depth, 3 = obstacle to roots between 40 and 60 cm depth, and/or 4 = obstacle to roots between 0 and 40 cm depth; BDGSF 1998^c^).

**Earthworm (ind./m²)** = Predicted median earthworm abundance from Rutgers et al. 2016^d^.

**Pasture (%)** = Percentage of permanent pasture surface (RPG 2012^e^).

**Maize (%)** = Percentage of maize crop surface (RPG 2012^e^).

**Urban. (%)** = Percentage of urbanized area (classes: 11; 121; 123; 124; CORINE Land Cover 2015^f^).

**Road (km/km²)** = Road density including main and secondary roads (IGN 2015^a^).

^a^: IGN BD Topo 2015, French Institute of Geography, [www.ign.fr](http://www.ign.fr)

^b^: IGN BD Alti 2011, French Institute of Geography, [www.ign.fr](http://www.ign.fr)

^c^: BDGSF 1998, “Base de Données Géographique des Sols de France”, [www.gissol.fr](http://www.gissol.fr)

^d^: Digital soil mapping from habitat-response models (Rutgers et al. 2016)

^e^: RPG 2012, French Ministry of Agriculture, [www.geoportail.gouv.fr](http://www.geoportail.gouv.fr)

^f^: CORINE Land Cover 2015, European Environment Agency, [www.eea.europa.eu](http://www.eea.europa.eu)

**Fig. S4.1.** Distribution of distances (*minDistT*) between all the 533 setts found during the walked transect surveys on the 13 study sites (interval = 100 m).


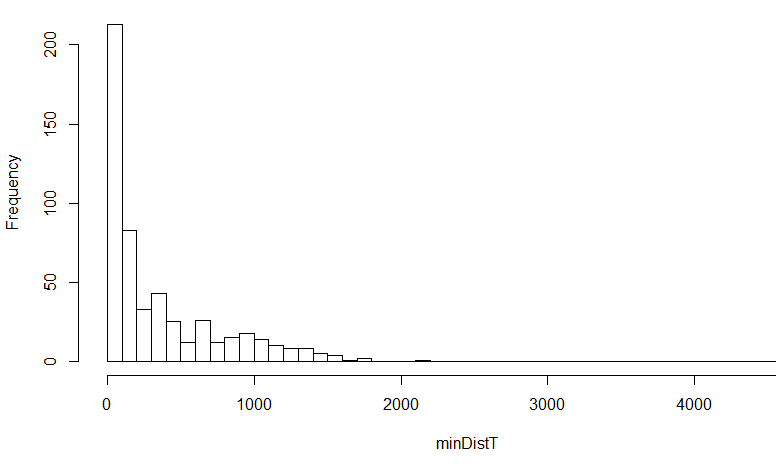


**Fig. S4.2.** Geographic maps of study sites D and I, with open areas (*white*), forest (*green*), forest edges of 50 m wide (*light green*) and hedgerows (*brown*) from BD TOPO Vegetation 2015 (IGN^a^). Using a systematic sampling method, theoretical transects (red lines) were placed in close vicinity to each theoretical point (black dots spaced 1 km apart) in suitable habitats for badger settlement.

^a^: IGN BD Topo 2015, French Institute of Geography, [www.ign.fr](http://www.ign.fr)

**Study site D.**


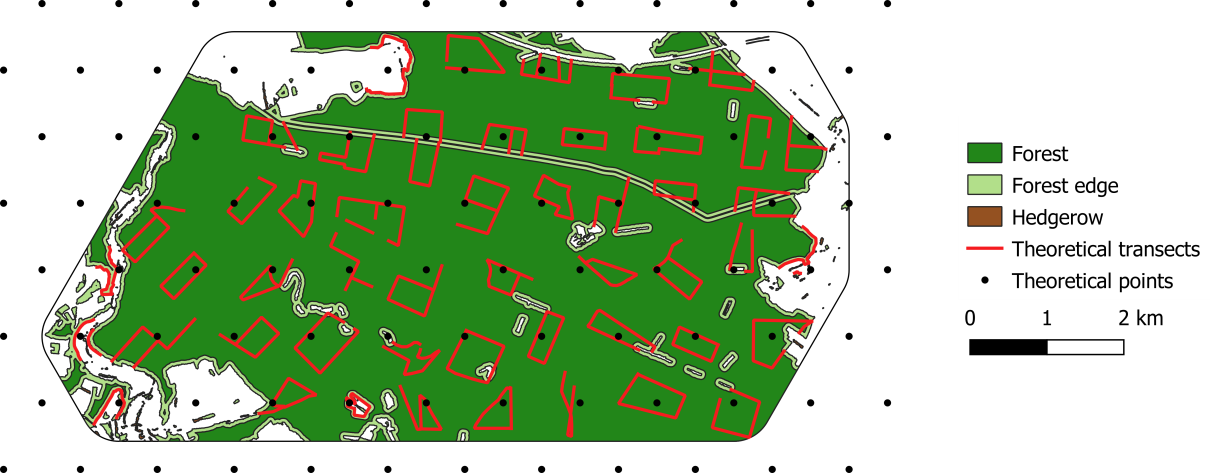


**Study site I.**


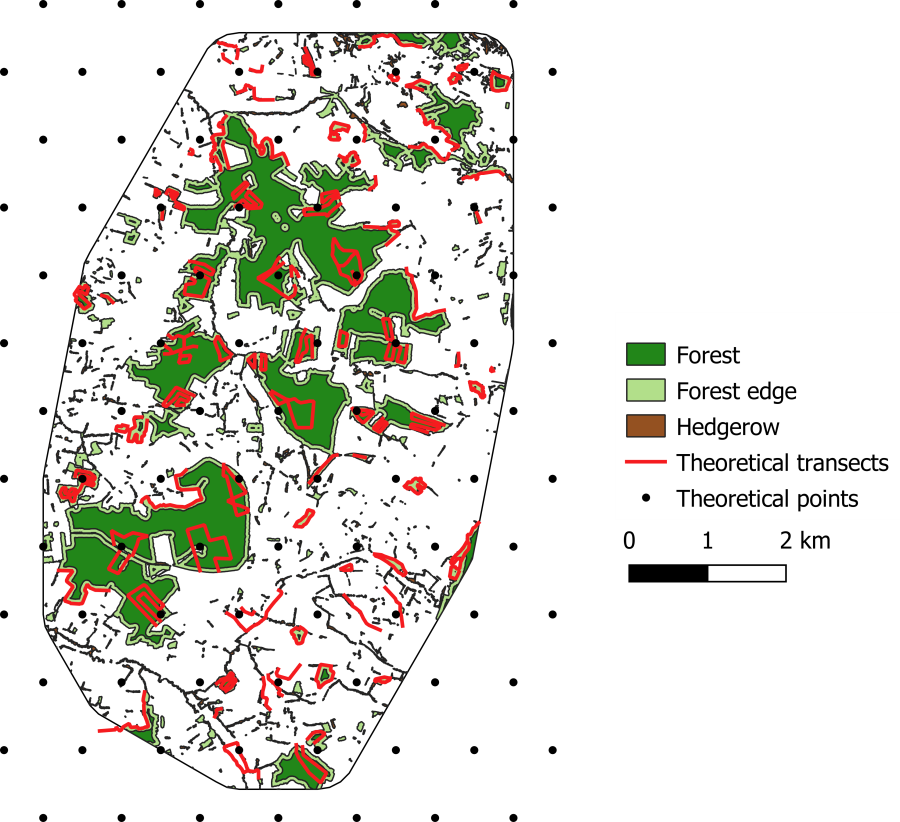


**Fig. S4.3.** Geographic maps of the 13 study sites spread throughout France (from A to M).

On each following page, we provide geographic maps of the 13 study sites spread throughout France (from A to M), with open areas (*white*), forest (*green*), forest edges of 50 m wide (*light green*) and hedgerows (*brown*) from BD TOPO Vegetation 2015 (IGN^a^). Two maps per site with a scale of 1/75 000, representing first the walked transect survey (red lines), and second, the discovered badger setts (black points) with 250 m-radius buffers representing badger sett clusters (red circles).

^a^: IGN BD Topo 2015, French Institute of Geography, [www.ign.fr](http://www.ign.fr)

**Study site A.**

**
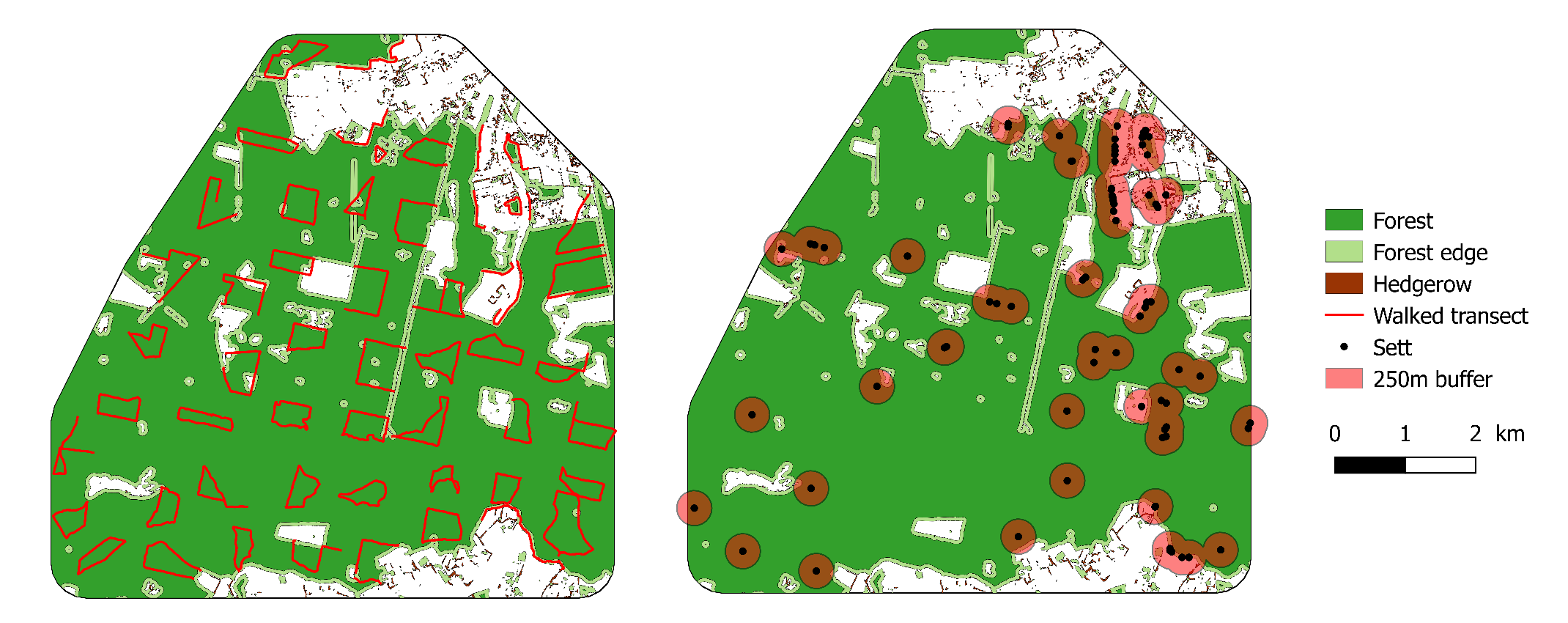
**

**Study site B.**

**
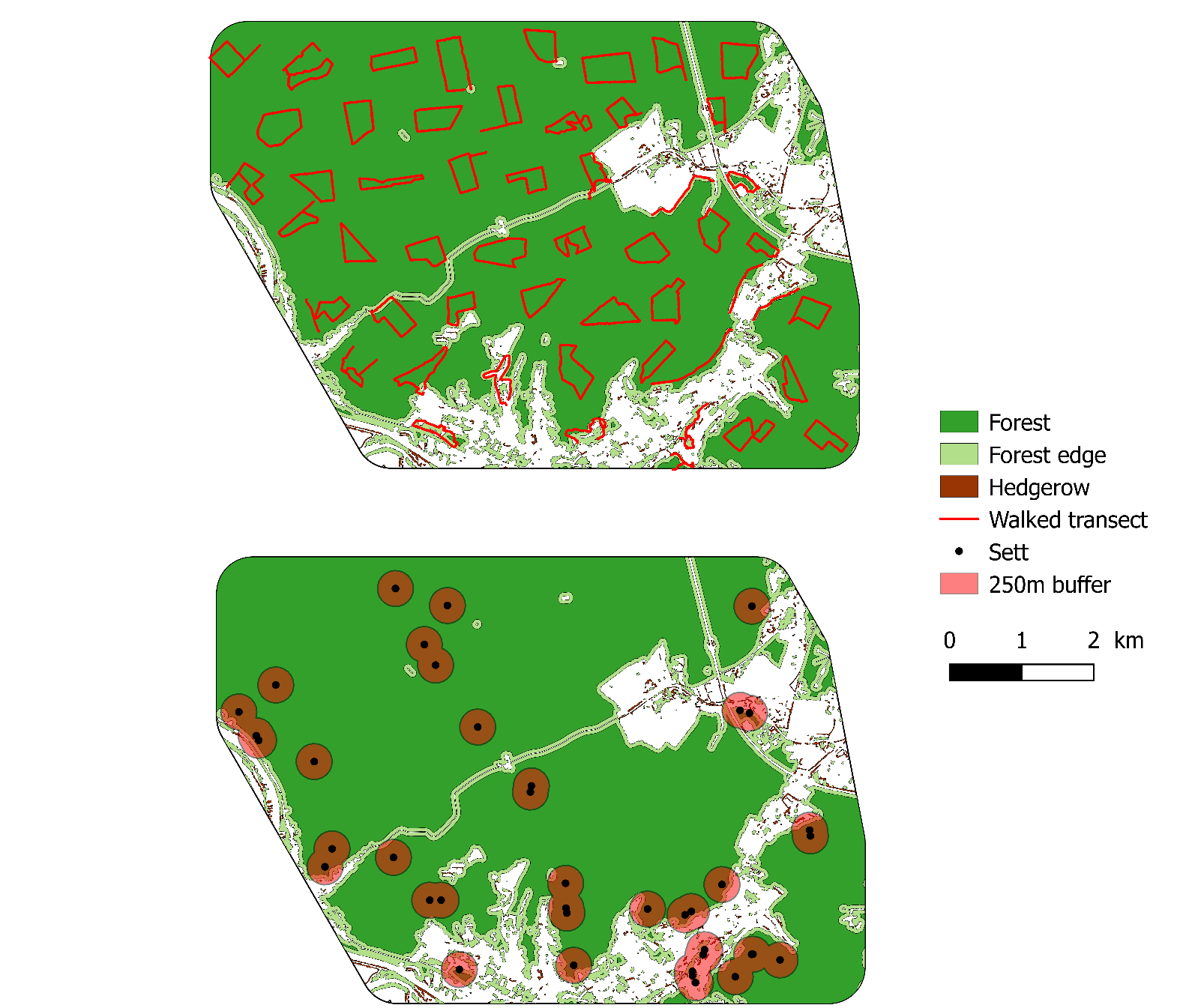
**

**Study site C.**

**
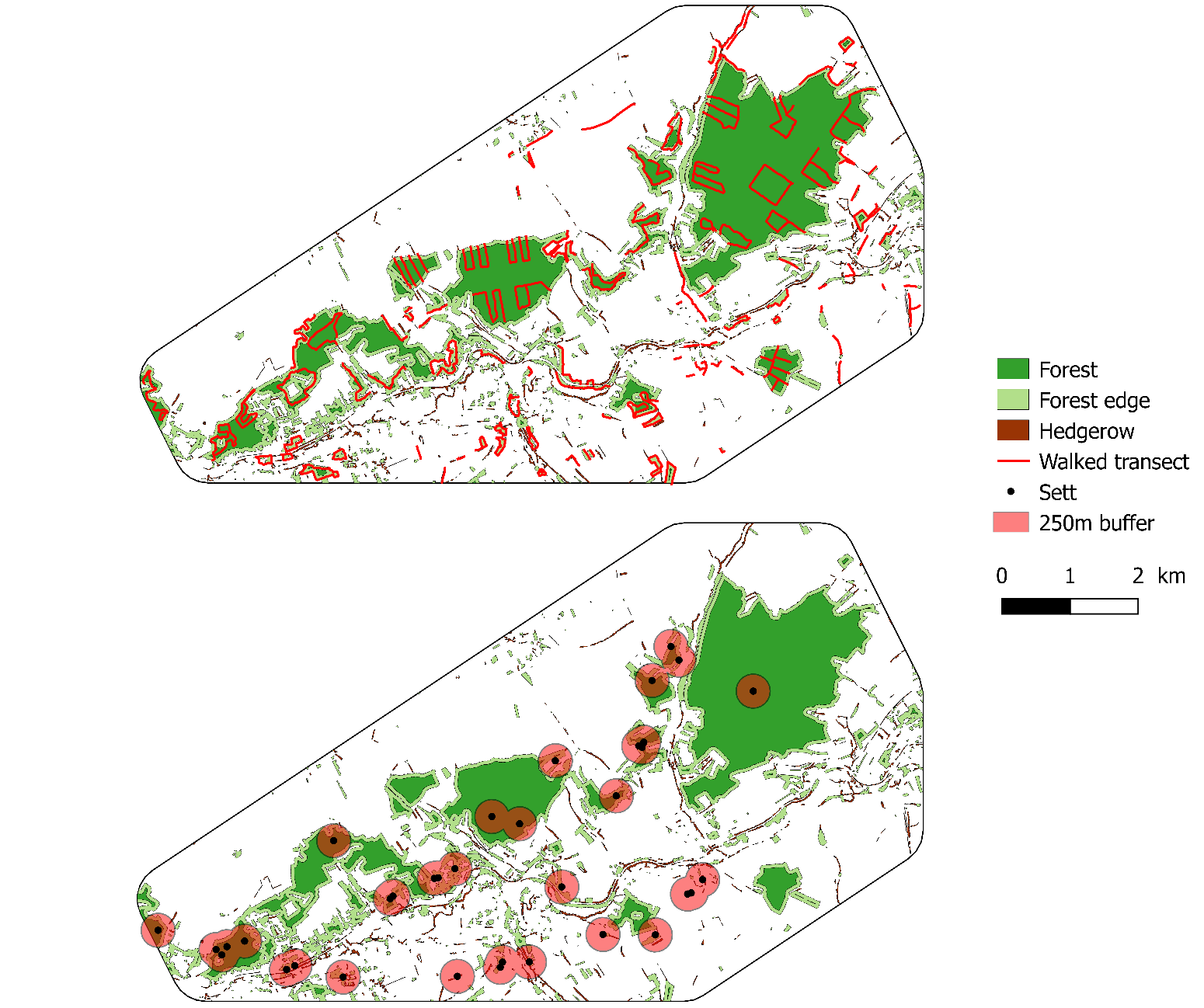
**

**Study site D.**

**
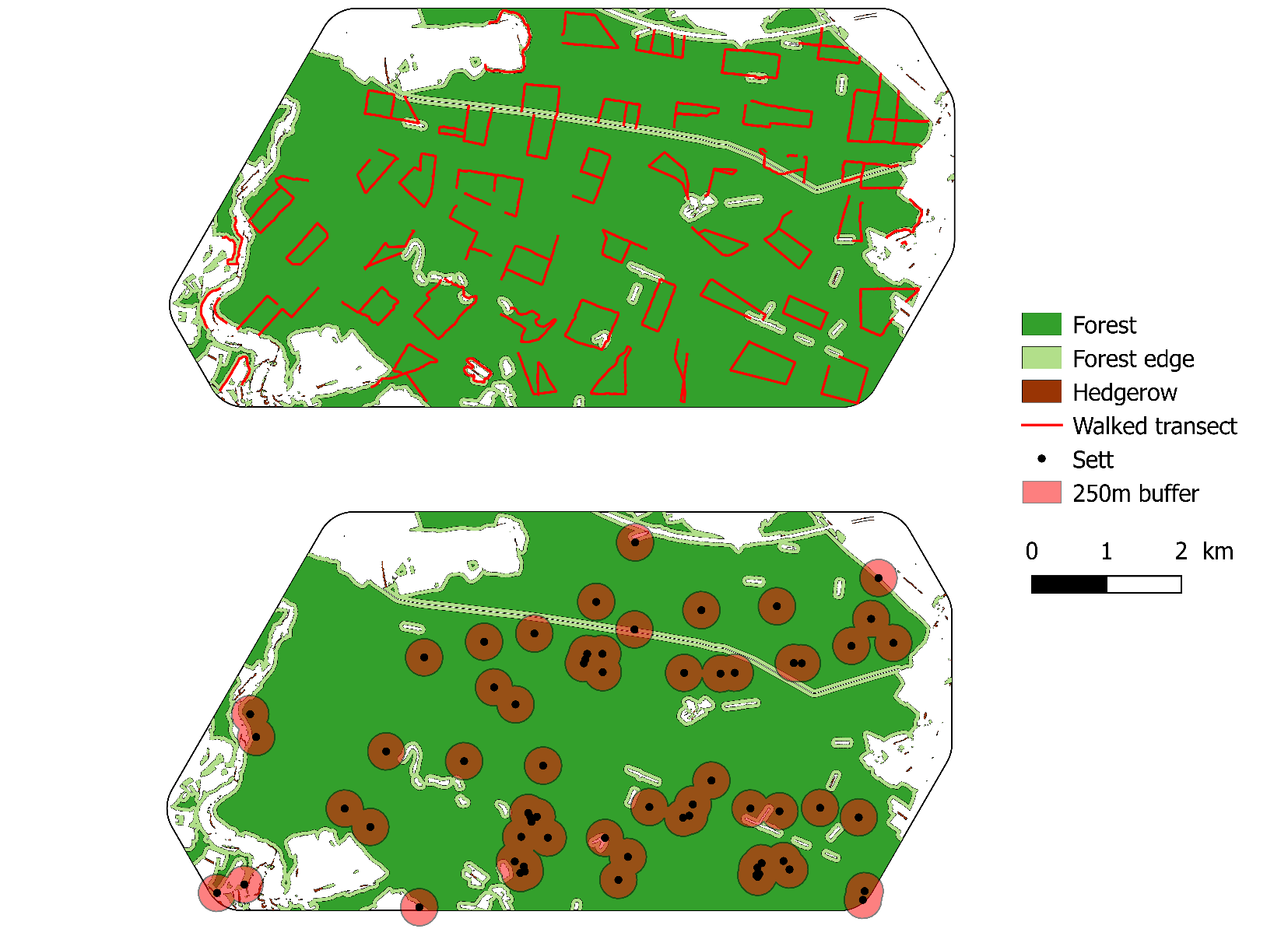
**

**Study site E.**

**
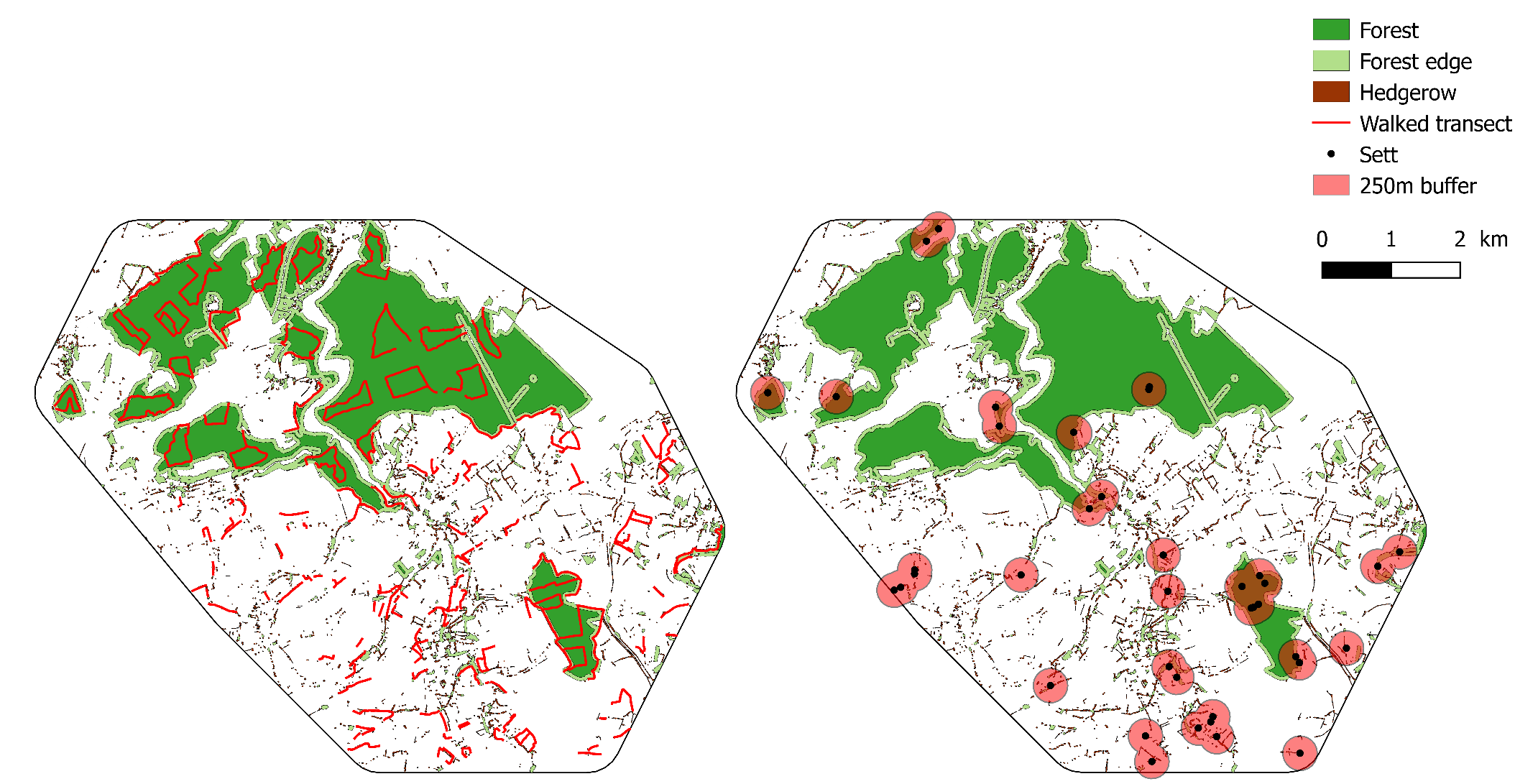
**

**Study site F.**

**
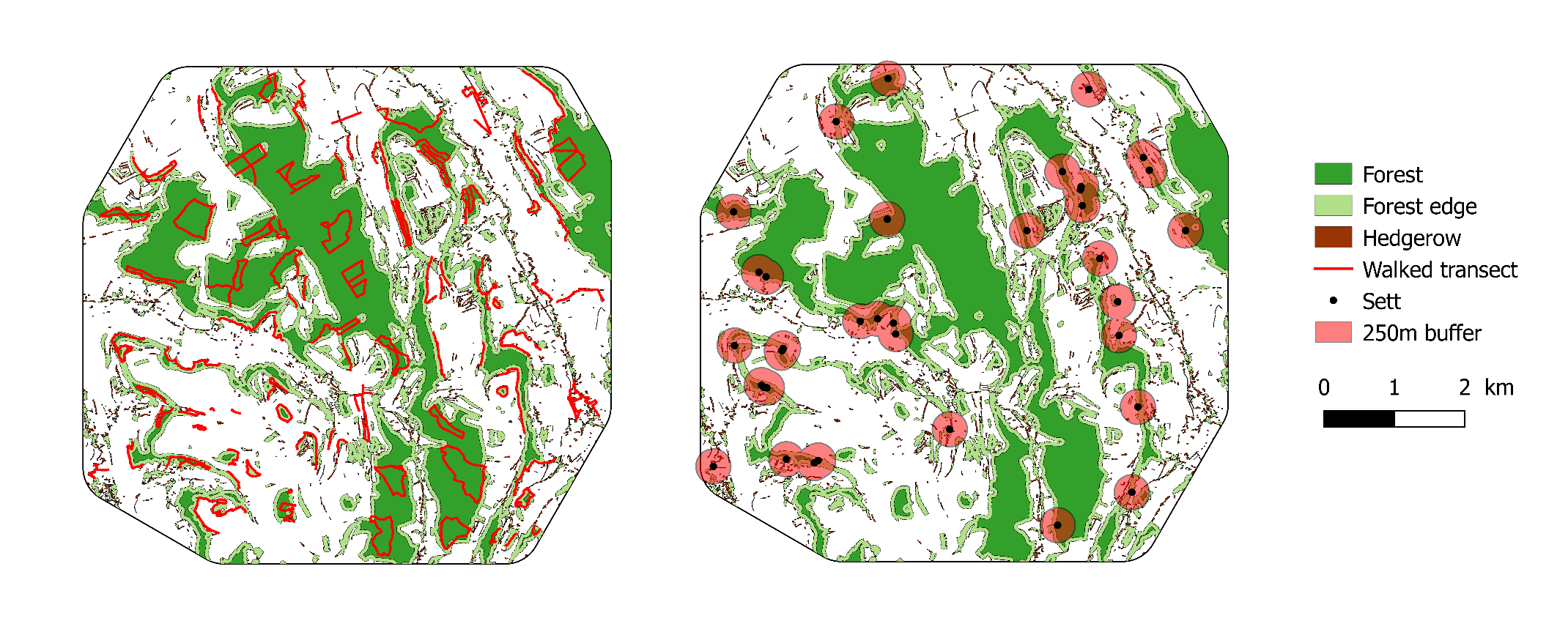
**

**Study site G.**

**
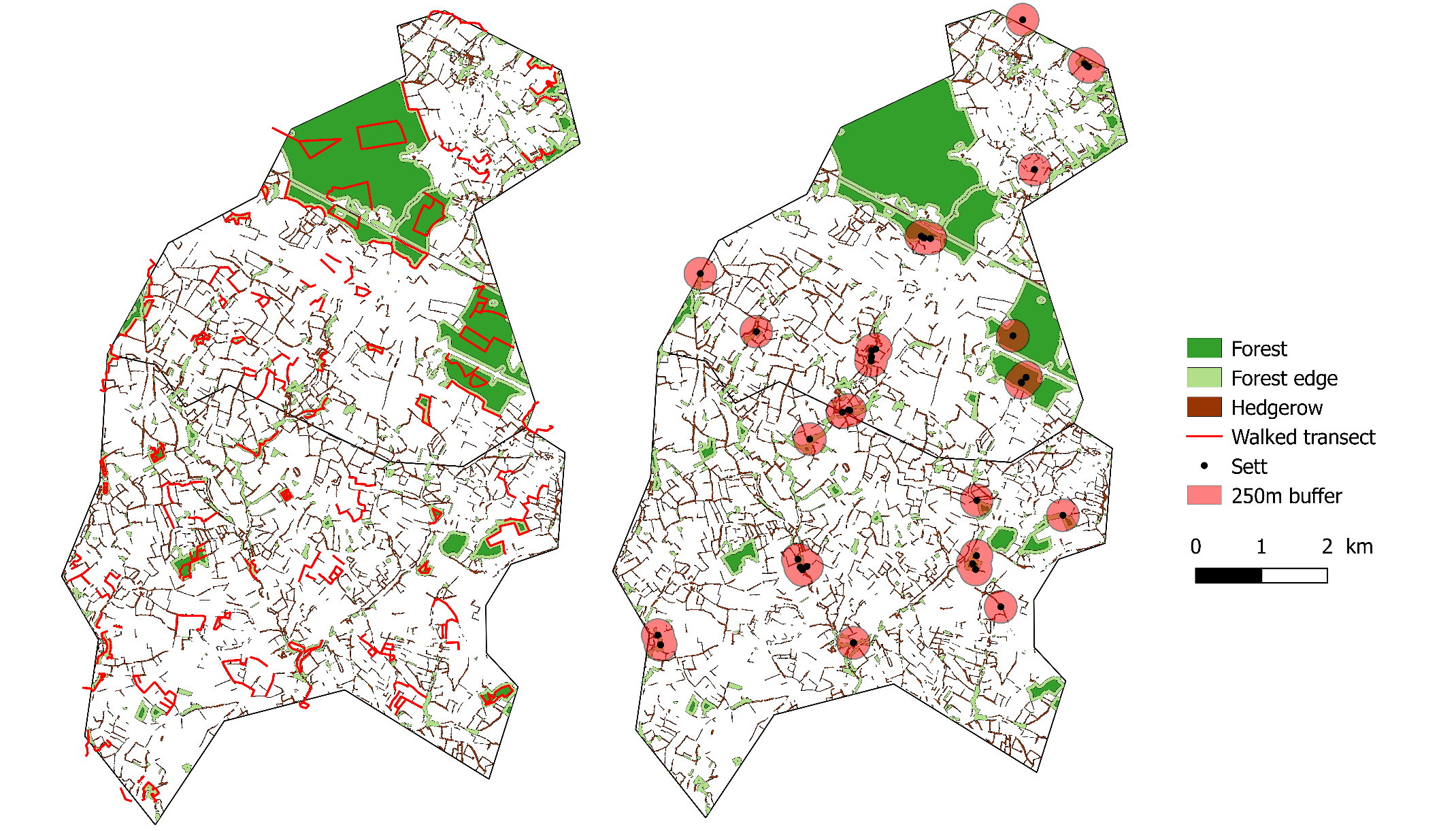
**

**Study site H.**

**
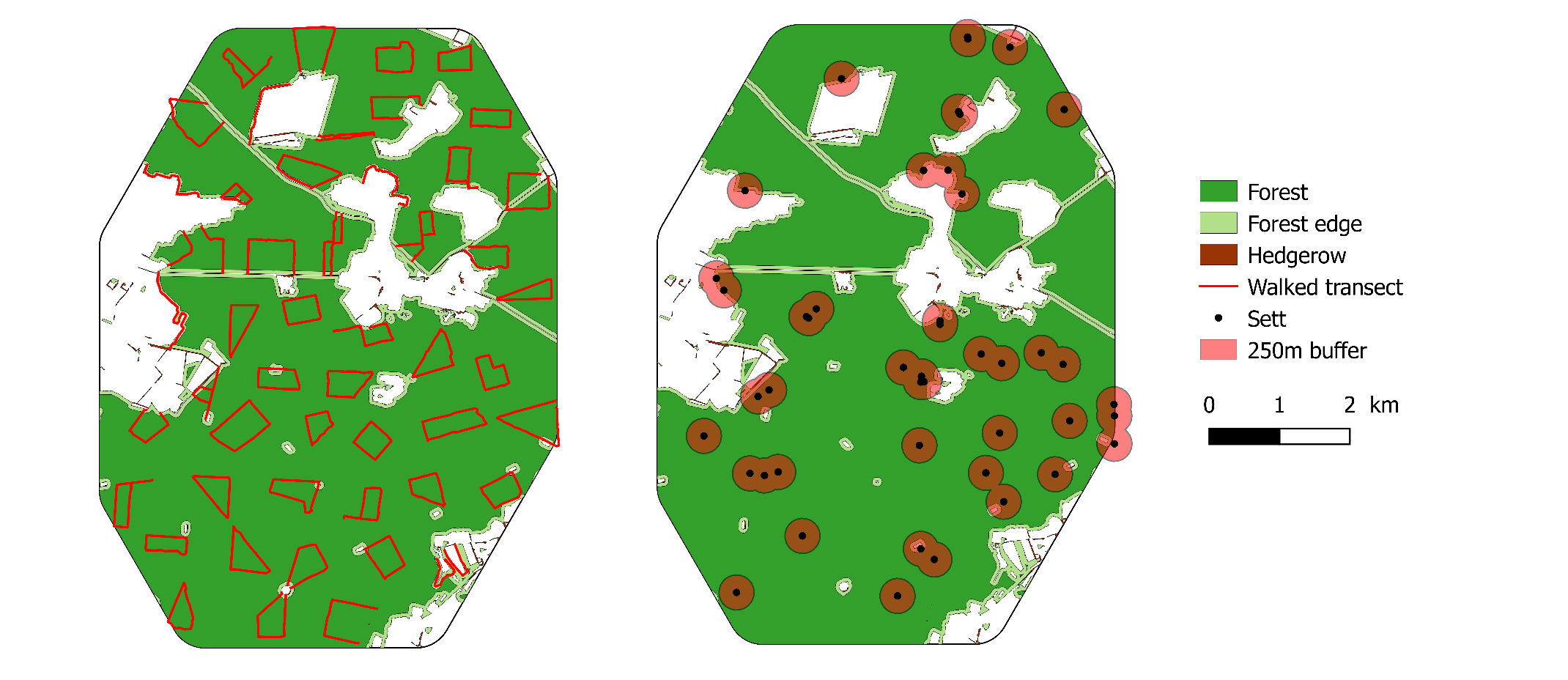
**

**Study site I.**

**
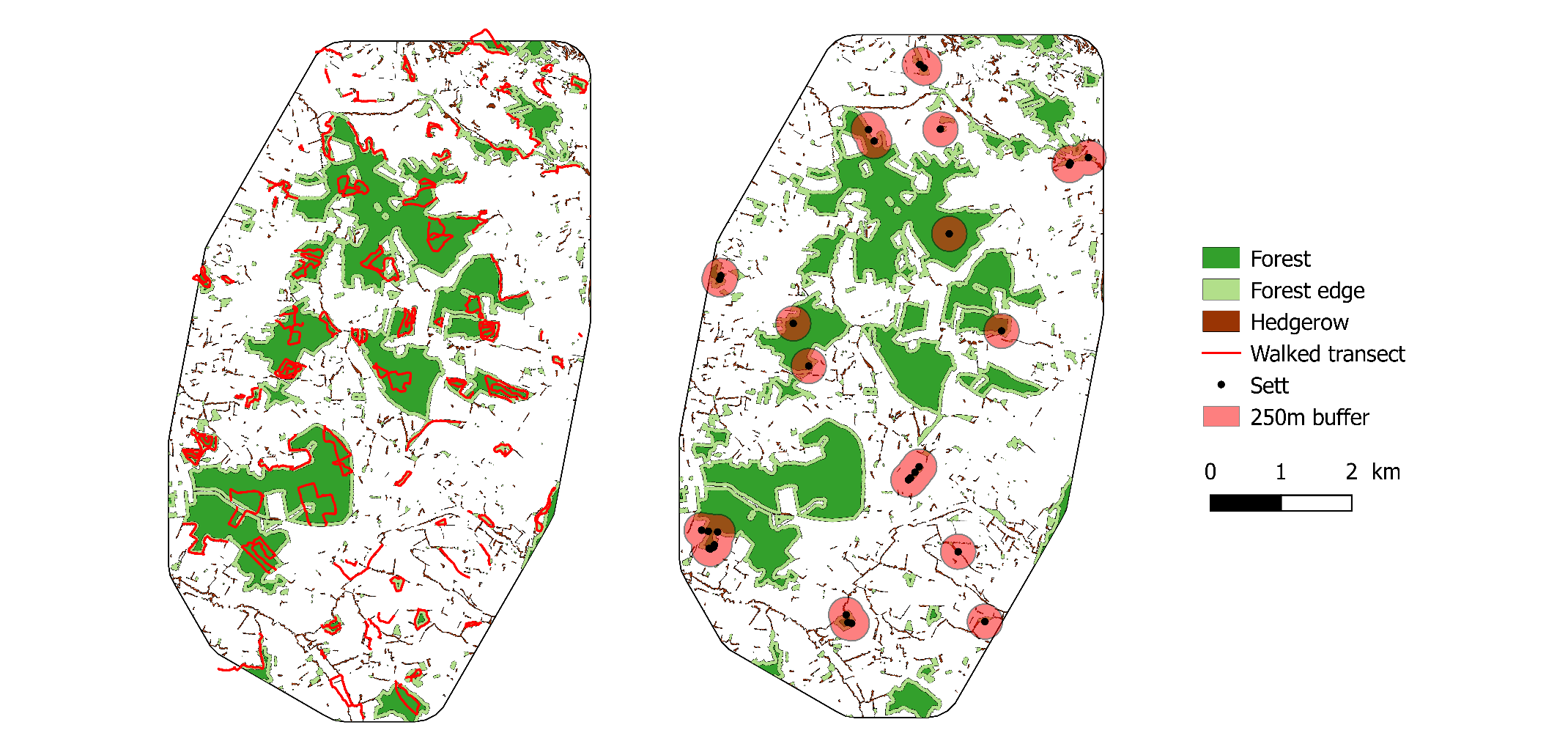
**

**Study site J.**

**
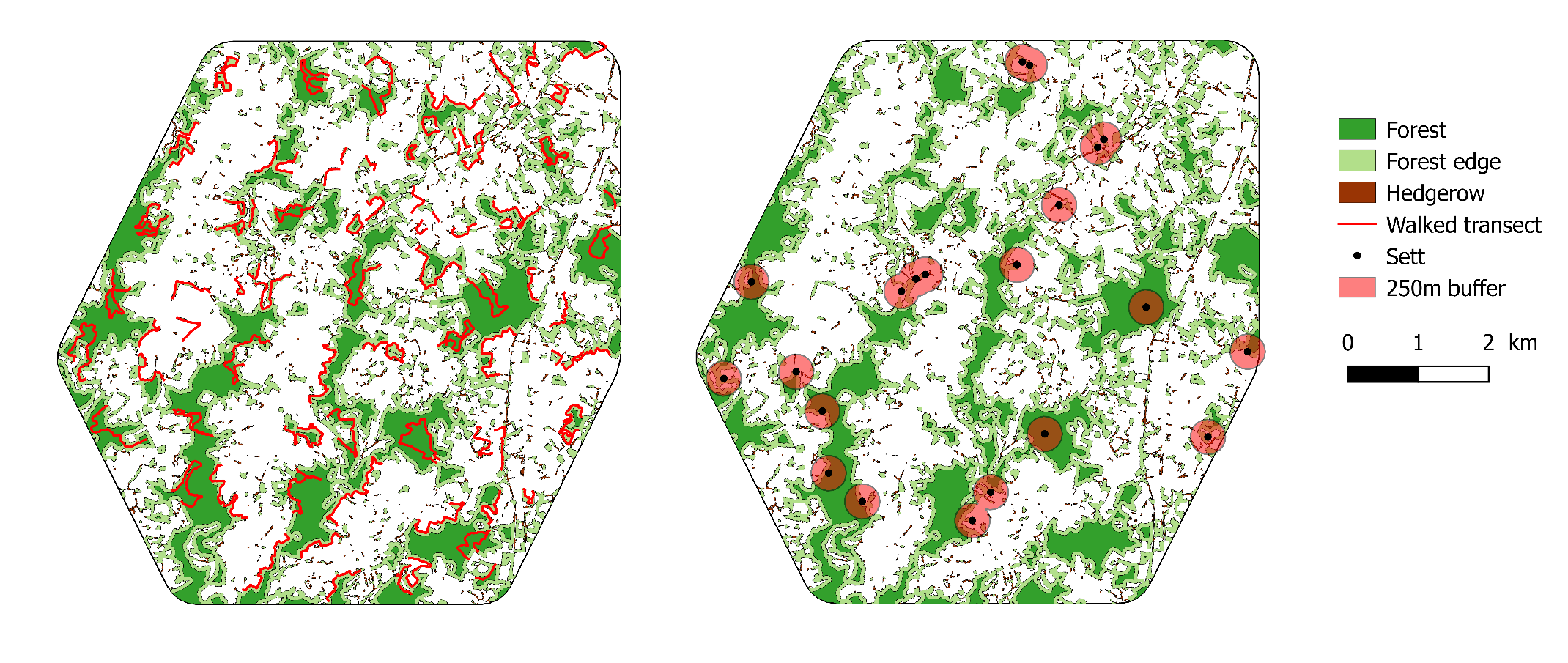
**

**Study site K.**

**
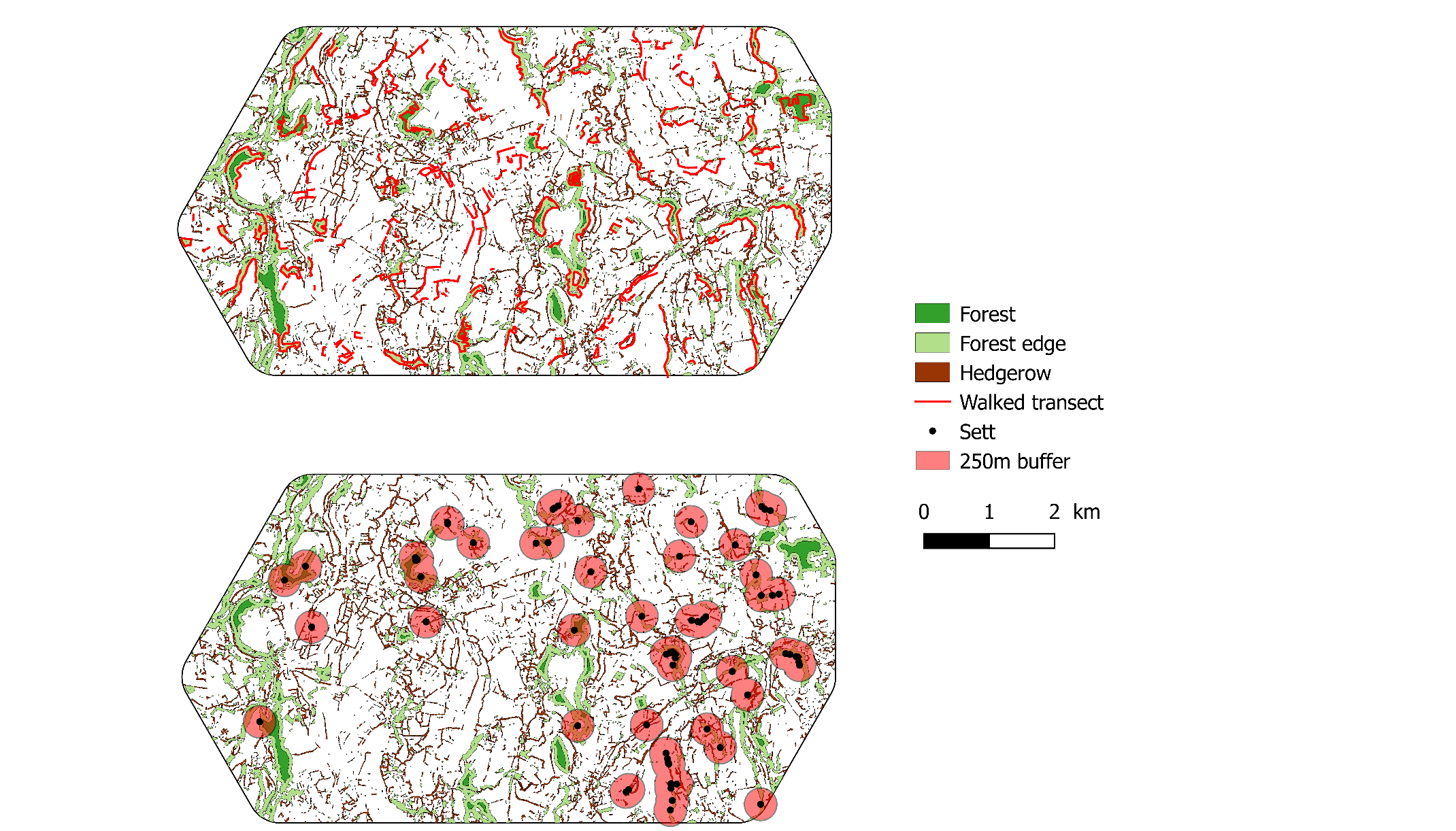
**

**Study site L.**

**
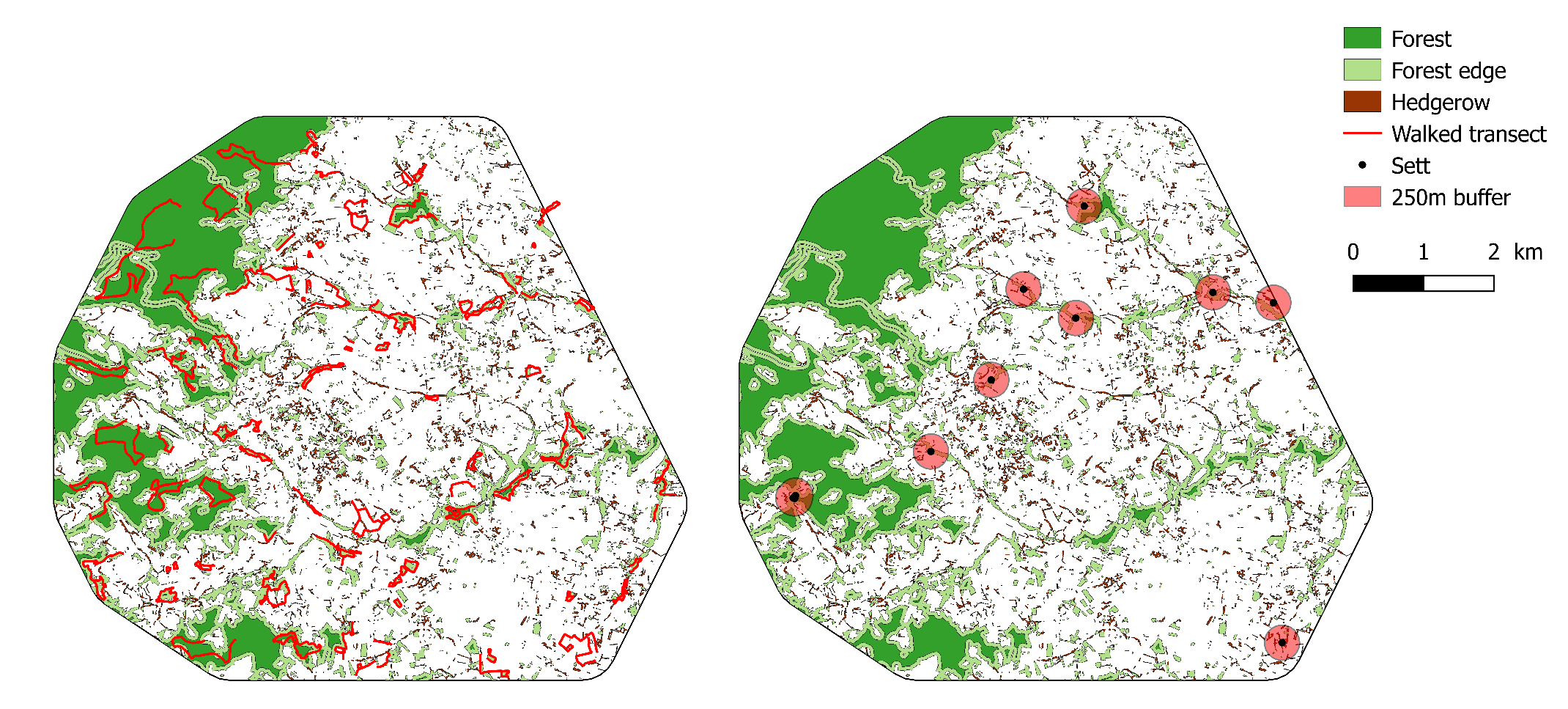
**

**Study site M.**

**
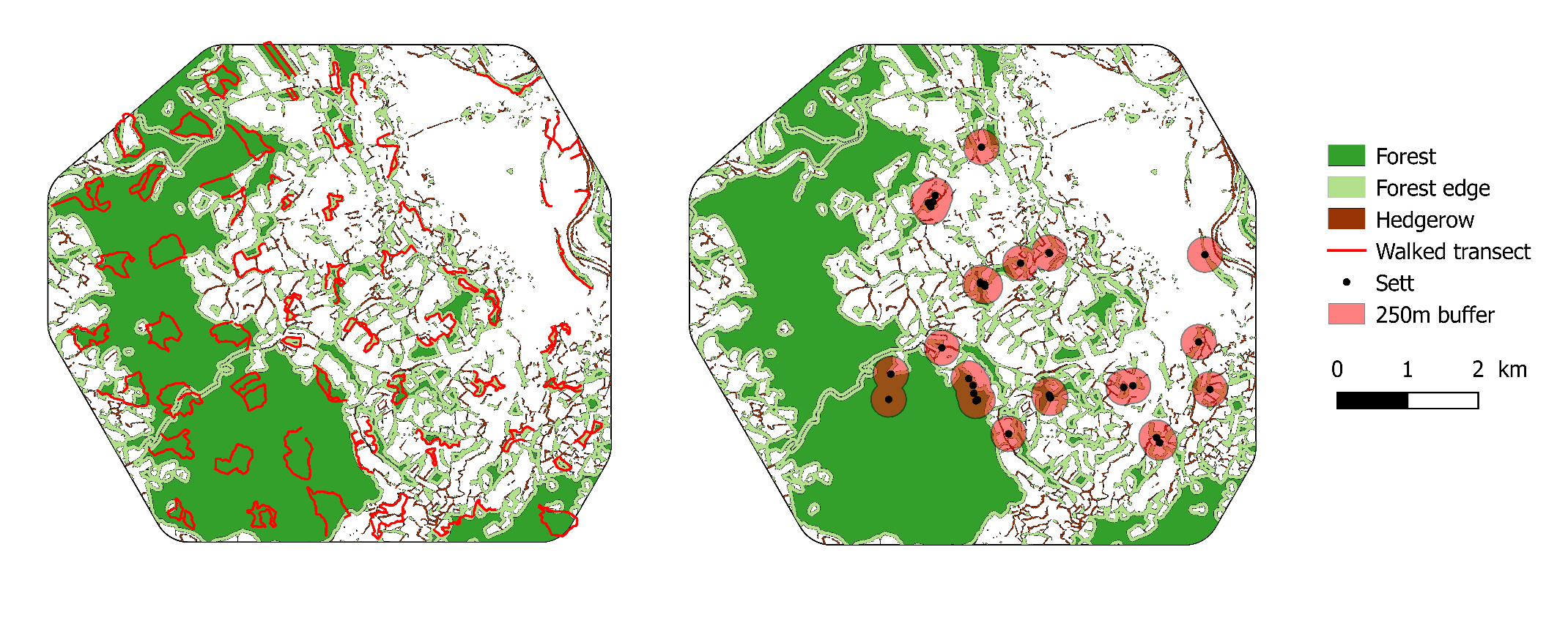
**
