# Supplementary material for: Breaking down population density into different components to better understand its spatial variation
Source: BMC Ecol Evol. 2021 May 11;21:82. doi: 10.1186/s12862-021-01809-6 (PMC8111954; doi:10.1186/s12862-021-01809-6)
Supplement: Supplementary file 5 — Additional file 5. Standardized scoring system used in order to distinguish badger main setts, occupied secondary setts, and unoccupied setts across all study sites. [file 12862_2021_1809_MOESM5_ESM.docx]

**Additional file 5**

This supplementary describes the standardized scoring system used in order to distinguish main setts, occupied secondary setts, and unoccupied setts across all study sites. This method allows for controlling for observer bias in sett classification.

Firstly, we performed a Multiple Correspondence Analysis (MCA; Fig. S5.1) on 14 categorical variables recorded on 650 setts in April during a 5-days field session to assess sett activity (i.e. different type of activity signs; described in the Table S5.1). The database included all setts present in the main text of this manuscript (i.e. = 533), and some other setts out of the protocol (e.g. known but not discovered during the walked transects survey; n = 117). We performed the MCA only considering the 594 setts, among the 650 in total, for which all 14 variables were described. The statistical analysis was done using the *FactoMineR* package [1] operating in R software [2].

**Table S5.1.** Categorical variables (n = 14) used to perform the Multiple Correspondence Analysis.

| **Abbreviation** | **Description** | **Categories** |
| --- | --- | --- |
| THAB | Transect's habitat | Forest |
|  |  | Forest edge |
|  |  | Hedgerow |
| SHAB | Sett's habitat | Stump |
|  |  | Rocks |
|  |  | Embankment |
| SLP | Slope at the sett | flat (0%) |
|  |  | weak (<5%) |
|  |  | medium |
|  |  | high (>20%) |
| HO | Total number of hole entrances | 1 |
|  |  | 2 ≤ x ≤ 5 |
|  |  | x ≥ 6 |
| UHO | Number of used hole entrances | 0 |
|  |  | 1 |
|  |  | 2 ≤ x ≤ 5 |
|  |  | x ≥ 6 |
| PRU | Proportion of used hole entrances | 0 |
|  |  | x < 0.5 |
|  |  | x ≥ 0.5 |
| FEC | Presence of faeces in latrines | Yes |
|  |  | No |
| LIT | Presence of litter | Yes |
|  |  | No |
| PRI | Presence of prints, scratches on trunks, hair | Yes |
|  |  | No |
| LAT | Number of latrines | 0 |
|  |  | 1 ≤ x ≤ 2 |
|  |  | x ≥ 3 |
| EXC | Number of fresh excavated zones | 0 |
|  |  | 1 |
|  |  | x ≥ 2 |
| PLA | Presence of grooming or playing zones | Yes |
|  |  | No |
| PINT | Number of badger path on the sett (internal) | 0 |
|  |  | 1 |
|  |  | x ≥ 2 |
| PEXT | Number of badger patch at sett's periphery (external) | 0 |
|  |  | 1 ≤ x ≤ 2 |
|  |  | x ≥ 3 |

**Fig. S5.1.** Contributions of the 14 variables and their categories to each dimension of the MCA.


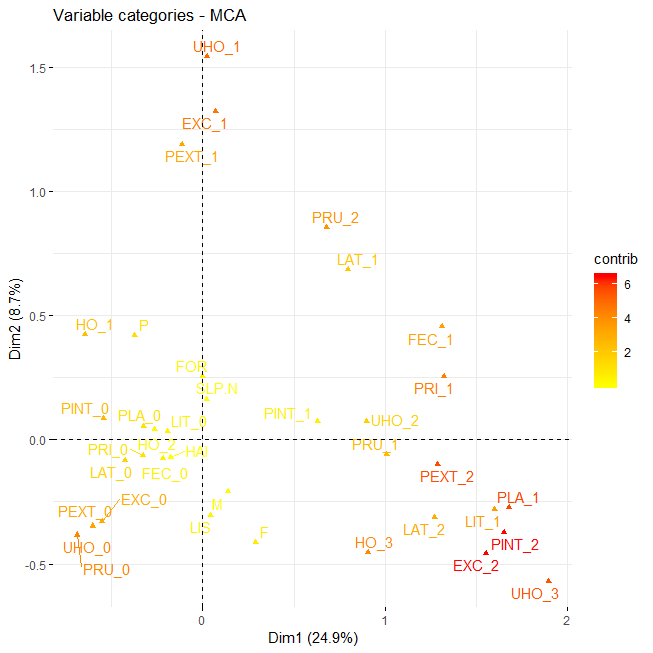


The two first dimensions of the MCA explained 33.6% of the total variance in the data (contribution of 24.9% and 8.7% for dimensions 1 and 2 respectively). The categories “1” for PLA and PRI, “2” for PINT, EXC and PEXT, and “3” for UHO have an important contribution to the positive pole of the first dimension, while the categories “1” for UHO, EXC and PEXT have a major contribution to the positive pole of the second dimension (Fig. S5.1; Fig. S5.2).

**Fig. S5.2.** Bar plots of the variable category contributions for each MCA dimensions (Dim1 and Dim2). The red dashed line indicates the expected average value under uniform contributions.


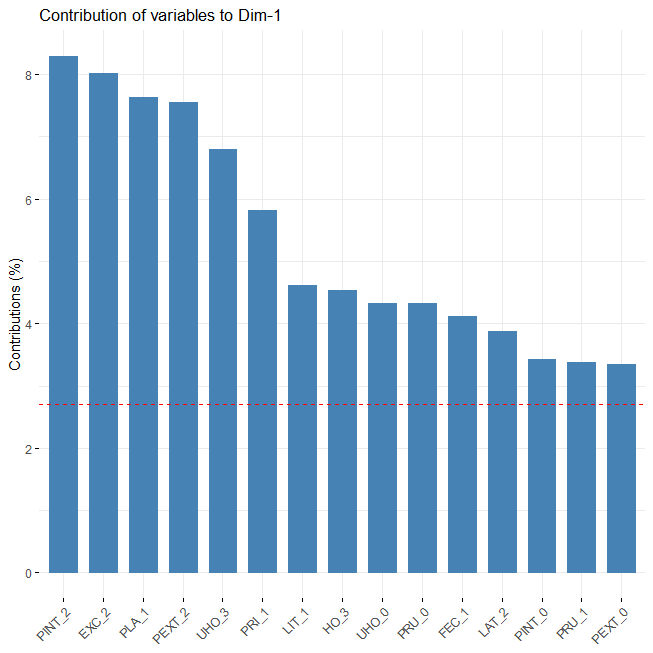

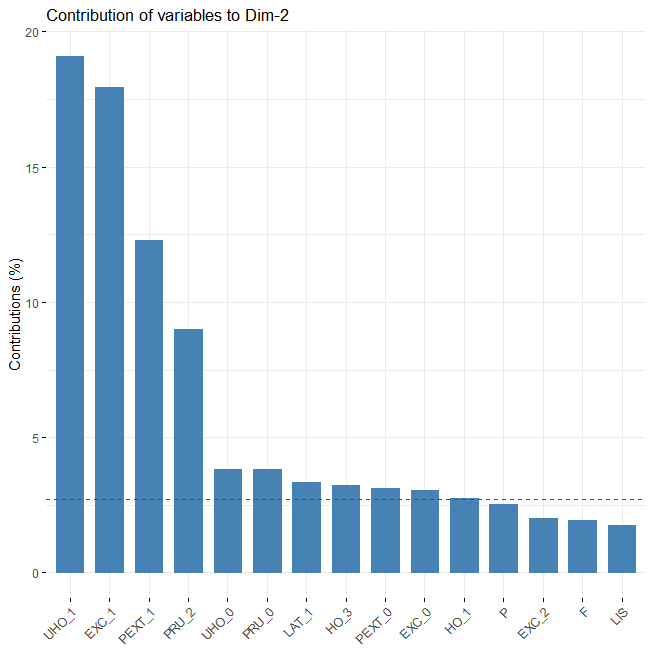


Secondly, based on these 6 variables that best discriminate setts (namely UHO, EXC, PLA, PRI, PINT, PEXT), we developed a scoring system to classify the 594 setts in either (i) unoccupied setts (score =0), (ii) occupied secondary setts (scores between 1 and 4) or (iii) main setts (score above 4). Theses scores were obtained by summing points according to the criteria described in Table S5.2 (see the graphical representation in Fig. S5.3). We also calculated scores for the 56 setts for which some variables were not recorded, by attributing the mean value of the considered variable for any missing data (blue dots in Fig. S5.3).

The accuracy of our scoring system to distinguish main, from secondary or unoccupied setts was tested by discriminant analysis. We found that the vast majority of the 594 setts were well grouped with the discriminant analyses compared to their attributed scores (99%, 74%, and 90% of the setts classified as unoccupied, secondary or main setts respectively). Our scoring method thus seem to be a powerful approach to categorise setts, and can be easily implemented in the field by only monitoring 6 activity signs (instead of 14 here initially).

**Table S5.2.** Attribution of points to established an occupational score for each sett, depending on the six main signs of activity.

| **Points attributed to each**  **activity sign** | **+0 point** | **+1 point** | **+2 points** |
| --- | --- | --- | --- |
| UHO: Used hole entrances | 0 | 1 | ≥ 2 |
| EXC: Fresh excavations | 0 | 1 | ≥ 2 |
| PINT: Internal badger path | 0 | 1 | ≥ 2 |
| PEXT: External badger path | 0 | 1 or 2 | ≥ 3 |
| PLA: Grooming or playing zones | None | Presence of a grooming zone | Presence of a playing zone |
| PRI: Prints or scratched trunks | None | Presence of prints and/or scratches on trunks |  |

**Fig. S5.3.** Biplot of variable categories on each dimension of the MCA, and the individuals (here 594 setts) depending of their score (i.e. unoccupied setts in black, secondary setts in yellow and main setts in red). Setts with incomplete description (n = 56) are represented with blue dots.


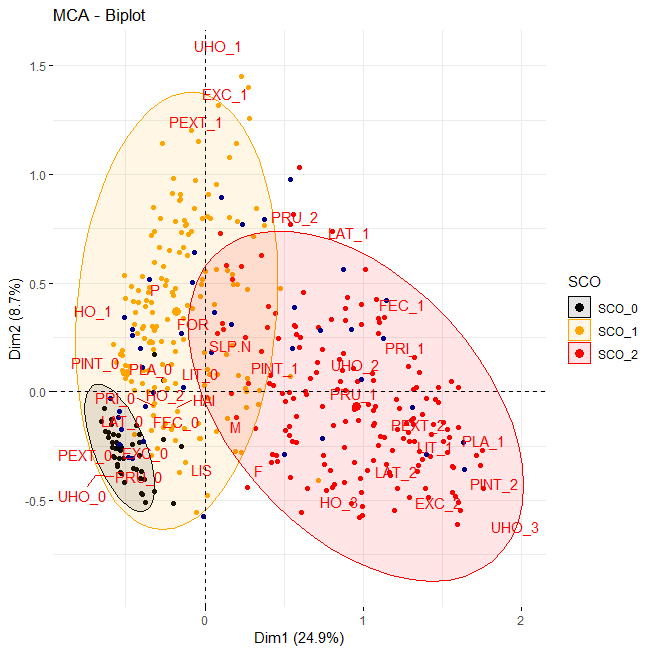


**Reference**

1. Lê S, Josse J, Husson F. FactoMineR: an R package for multivariate analysis. J Stat Softw. 2008;25:1–18.

2. R Development Core Team 2019. R: a language and environment for statistical computing. R Foundation for Statistical Computing, Vienna, Austria. http://www.r-project.org/
